# Predictive validity of daily sequential organ failure assessment (SOFA)-2 score for 30-day mortality

**DOI:** 10.1186/s13054-026-06093-8

**Published:** 2026-06-01

**Authors:** Johan Helleberg, Anna Sundelin, Navid Soltani, Olav Rooyackers, Johan Mårtensson

**Affiliations:** 1 Department of Perioperative Medicine and Intensive Care, Karolinska University Hospital, Stockholm, Sweden; 2 Section of Anaesthesiology and Intensive Care Medicine, Department of Clinical Sciences, Intervention and Technology (CLINTEC), Karolinska Institutet, Stockholm, Sweden; 3 Department of Physiology and Pharmacology, Section of Anaesthesia and Intensive Care Karolinska Institutet, Karolinska Institutet, 171 77 Stockholm, Sweden

**Keywords:** SOFA score, Organ failure, Critical illness, Mortality

## Abstract

**Purpose:**

The sequential organ failure assessment (SOFA) score was recently updated to better reflect contemporary intensive care. This study compares the predictive validity of the updated SOFA-2 and the previous SOFA-1 for 30-day mortality over the first week in ICU, overall and in subgroups with distinct age and comorbidity profiles.

**Methods:**

We conducted a retrospective observational study of adult patients admitted to four ICUs in Sweden from 2010 to 2021. Predictive validity for 30-day mortality was assessed on day 1–7 using the area under the receiver operating characteristic curve (AUROC).

**Results:**

We included 29,820 admissions (mean age 60 years, 64.8% males, median Charlson comorbidity index [CCI] 1). Reclassification between SOFA-1 and SOFA-2 occurred in 75–79% of admissions across days 1–7. On day 1, the AUROC for 30-day mortality was 0.81 (95% CI 0.80–0.81) for SOFA-2 and 0.80 (95% CI 0.79–0.81) for SOFA-1 (p < 0.001). From day 2 onward, AUROCs declined for both scores and were largely similar. Among trauma patients (mean age 50 years; median CCI 0), day-1 AUROC for SOFA-2 was 0.81 (95% CI 0.79–0.83), while among sepsis patients (mean age 61 years; median CCI 3) it was 0.72 (95% CI 0.70–0.74), with comparable performance for SOFA-1.

**Conclusions:**

SOFA-2 provided modestly better discrimination for 30-day mortality on ICU-day 1 compared with SOFA-1. Predictive validity diminished over subsequent days for both scores and varied across subgroups with different age and comorbidity distributions, underscoring the context-dependence of organ dysfunction scoring.

**Trial registration:**

https://doi.org/10.5281/zenodo.17651826, registration date November 19, 2025.

**Supplementary Information:**

The online version contains supplementary material available at 10.1186/s13054-026-06093-8.

## Introduction

The sequential organ failure assessment (SOFA) score has guided the evaluation of organ dysfunction and illness severity in critically ill patients for more than two decades. However, the original SOFA-1 score was derived from 1990s-era data [1, 2], before major advances in organ supportive therapies. The recently proposed SOFA-2 score [3] represents a major recalibration derived from over three million contemporary ICU admissions. While SOFA-2 showed similar overall discrimination for ICU mortality compared with SOFA-1, it provided a more consistent mapping between modern treatment practices and graded organ failure.

Despite the scale and methodological rigor of the SOFA-2 derivation and validation, several uncertainties remain [4]. Most importantly, SOFA-2 was validated against ICU mortality, an outcome influenced by discharge practices and resource constraints and one that fails to capture deaths occurring after ICU discharge. This limits comparability with clinical trials and prognostic models that commonly use 28- or 30-day mortality. Moreover, although the study reported daily SOFA-2 patterns during the first ICU week in a subgroup of mainly US admissions, it did not assess time-dependent calibration or conditional mortality. These elements are central to interpreting organ-failure dynamics and to determining whether SOFA-2 provides clinically coherent prognostic signals over time. Finally, the SOFA framework does not distinguish chronic from acute organ dysfunction; these conditions may follow different temporal patterns and may contribute unequally to short-term mortality risk.

To address these gaps, we performed an independent validation of SOFA-2 in a large cohort of adult ICU patients, using 30-day mortality as the primary outcome. Our aims were to compare daily SOFA-2 and SOFA-1 trajectories during the first ICU week, evaluate their reclassification behavior, and assess their predictive performance over time, both in the overall population and in subgroups expected to differ in age and comorbidity distributions.

## Methods

This study was approved by the Swedish Ethical Review Authority (approval number 2022–06005-01) with a waiver of informed consent. The study protocol was pre-registered at 10.5281/zenodo.17651826.

### Study design and patient selection

We conducted a retrospective cohort study of adult (≥ 18 years) patients admitted to four ICUs at the Karolinska University Hospitals (Solna site and Huddinge site) in Stockholm, Sweden between January 2010 and June 2021. We excluded patients without a valid social security number. To correctly assess daily SOFA scores in relation to ICU admission, we also excluded patients who were admitted from ICUs outside the participating hospitals.

### Data collection

We collected data for SOFA-2 and SOFA-1 calculations, ICU admission diagnoses, and demographic data from the electronic patient data monitoring system (PDMS) Centricity Critical Care (GE Healthcare, Chicago, IL). We collected comorbidity data (International Classification of Diseases, 10th Revision codes) and information on date of death from the hospital electronic health record system Take Care (CompuGroup Medical, Koblenz, Germany).

### SOFA-2 and SOFA-1 computations

A previously developed and manually validated algorithm for calculating SOFA-1 scores was used as the foundation for this study [5]. The algorithm was built using data extracted from the Karolinska PDMS and electronic health record (EHR) systems and includes supervised machine-learning–based anomaly detection steps to remove erroneous physiological or laboratory entries before score derivation [6]. The high temporal resolution of the underlying database enables score calculation at arbitrary time points. This algorithm was adapted to incorporate the updated variable definitions and thresholds required for SOFA-2.

The majority of SOFA-2 components, (including bilirubin, creatinine, platelets, GCS subscores, PaO₂/FiO₂ ratio, mechanical ventilation, urine output, and noradrenaline equivalents), were manually validated during the development of the SOFA-1 algorithm and are shared with SOFA-2 with only minor threshold modifications. Variables unique to SOFA-2, namely delirium-related medication, extracorporeal membrane oxygenation (ECMO), and renal replacement therapy (RRT), were not subject to the same manual validation. However, review of the extraction logic and data sources for these variables indicated high data quality consistent with the validated components. In the present study, we calculated daily SOFA-2 and SOFA-1 scores for each patient’s entire stay in the ICU. SOFA scores after discharge from the ICU were not calculated. The analyses were performed on available daily SOFA scores for days 1–7, computed in 24-h intervals since ICU arrival. Delta SOFA was calculated using two predefined approaches: (1) the absolute change in score on each day relative to day 1, and (2) the absolute change compared with the preceding day. Mean SOFA score was defined as the mean of the daily SOFA-scores across all ICU-days for each patient, and maximum SOFA score as the highest recorded SOFA score during ICU stay.

### Operational definitions

We used the Charlson comorbidity index (CCI), recently adapted to a Swedish setting [7, 8], to classify the following comorbidities descriptively: renal disease (including moderate or severe kidney disease), liver disease (including mild to severe liver disease), cardiovascular disease (including myocardial infarction, congestive heart failure, peripheral vascular disease, and cerebrovascular disease), pulmonary disease (including chronic obstructive pulmonary disease and other chronic pulmonary disease), and malignancy (including any malignancy, leukaemia, lymphoma, and metastatic cancer). Sepsis at admission was defined according to the sepsis-3 criteria [9], using the retrospective methods described by Seymour et al. [10]. To determine suspected infection, antibiotics labelled as prophylaxis, intraoperative antibiotics during elective surgery, and single doses of antibiotics were disregarded. Patients with onset of sepsis within a 24-h window from ICU admission were considered to have sepsis at admission.

### Outcomes

Primary outcome was all-cause mortality at 30 days from ICU admission. Secondary outcome was ICU mortality.

### Statistical analysis

Analyses were performed using R version 4.5.2 (R Foundation for Statistical Computing, Vienna, Austria). Categorical variables are reported as counts with percentages. Continuous variables are summarized as medians with interquartile ranges (IQRs) or means with standard deviations (SDs), as appropriate. Distribution of SOFA-2 and SOFA-1 scores, as well as reclassification patterns between the two scoring systems, are presented with proportions and corresponding 95% confidence intervals (CIs). To assess reclassification, daily SOFA scores were categorised in the following three groups: (1) SOFA-2 = SOFA-1 (reference group), (2) SOFA-2 > SOFA-1, and (3) SOFA-2 < SOFA-1. We fitted a logistic regression model with an interaction term between ICU-day and group to allow group effect to vary over time. Daily odds ratios (ORs) for 30-day mortality were then computed, comparing each reclassification group to the reference. Predictive validity for mortality was assessed by calculating the area under the receiver operating characteristic curve (AUROC) from univariate logistic regression models based on daily SOFA scores, mean SOFA scores, maximum SOFA scores, and delta SOFA scores. We used the DeLong method [11] to estimate 95% CIs for the AUROCs and to compare AUROCs. Calibration was assessed using Brier score, calibration slope and intercept. Optimism-corrected estimates and 95% confidence intervals were computed using bootstrap resampling [12]. As secondary measures of incremental model performance, we also calculated continuous net reclassification improvement (NRI) and the event- and non-event components of integrated discrimination improvement (IDI + and IDI-) from predicted probabilities from SOFA-2 and SOFA-1 based models. We also calculated AUROCs for daily domain-specific subscores. The interaction between ICU-day and OR for death per 1-point increase in subscore was assessed in a multivariable, logistic regression model with cluster robust standard error estimation. As a sensitivity analysis, we fitted generalized linear mixed-effects models with a logistic link and a random intercept per ICU admission to estimate conditional (within-admission) associations and to compare the prognostic information in subscores versus the total SOFA-2 score while accounting for repeated measures. We compared three models: (i) a null model with random intercept only, (ii) a model with SOFA-2 and time since admission and (iii) a model including time and the individual subscores. Model fit was compared using the Akaike Information Criterion (AIC) [13].

In the primary analyses, missing data for day 1 were handled using normal-value imputation for each organ-specific domain. For days 2–7, missing values were imputed using last-observation-carried-forward, consistent with the approach recommended in the original SOFA-2 study [3, 14]. Sensitivity analyses using multiple imputation with chained equations for missing data across days 1–7 were performed to assess robustness of the findings. We also conducted pre-specified exploratory subgroup analyses in patients admitted with sepsis and in those admitted after trauma, reflecting groups expected to differ in baseline age and comorbidity profiles. Finally, due to a relatively short length of stay and median SOFA-1 score on admission in the overall cohort, we conducted post-hoc subgroup analyses in patients with an admission SOFA-1 score ≥ 10, to assess performance in more severely ill patients. When multiple comparisons occurred in an analysis, CIs and p-values were Bonferroni-adjusted. P-values < 0.05 were considered statistically significant.

## Results

### Study population

A total of 32,211 adult ICU admissions were recorded in the PDMS between January 2010 and June 2021. We excluded 880 admissions without a social security number and 1,672 admissions from other ICUs. Therefore, we included 29,820 admissions (mean age 60 [SD 16] years, 64.8% males) in the final study cohort (Supplementary Figure S1). Median (IQR) CCI was 1 (0–3). Cardiovascular disease (44.1%) and malignancy (20.6%) were the most prevalent comorbidities. Overall, 17.5% had sepsis at admission and 10.0% were admitted following trauma. ICU and 30-day mortality was 8.9% and 14.3%, respectively (Table 1).

**Table 1:** Patient characteristics

Characteristic	All admissions
	(n = 29,820)
Age, mean (SD), year	60 (16)
Male sex, n (%)	19,333 (64.8)
Charlson comorbidity index, median (IQR)	1 (0–3)
Selected comorbidities, n (%)	
Renal disease	2,966 (10.0)
Liver disease	2,374 (8.0)
Cardiovascular disease	13,166 (44.1)
Pulmonary disease	4,494 (15.1)
Malignancy	6,145 (20.6)
ICU admission type, n (%)	
Medical	13,565 (45.5)
Elective surgical	10,671 (35.8)
Emergency surgical	5,584 (18.7)
Sepsis at ICU admission, n (%)	5,206 (17.5)
Admitted following trauma, n (%)	2,982 (10.0)
Invasive mechanical ventilation at ICU admission, n (%)	18,049 (60.5)
ICU length of stay, median (IQR), days	2 (1–4)
ICU mortality, n (%)	2,643 (8.9)
30-day mortality, n (%)	4,276 (14.3)

ICU, intensive care unit

### SOFA score distributions and reclassification characteristics

**Fig. 1:**
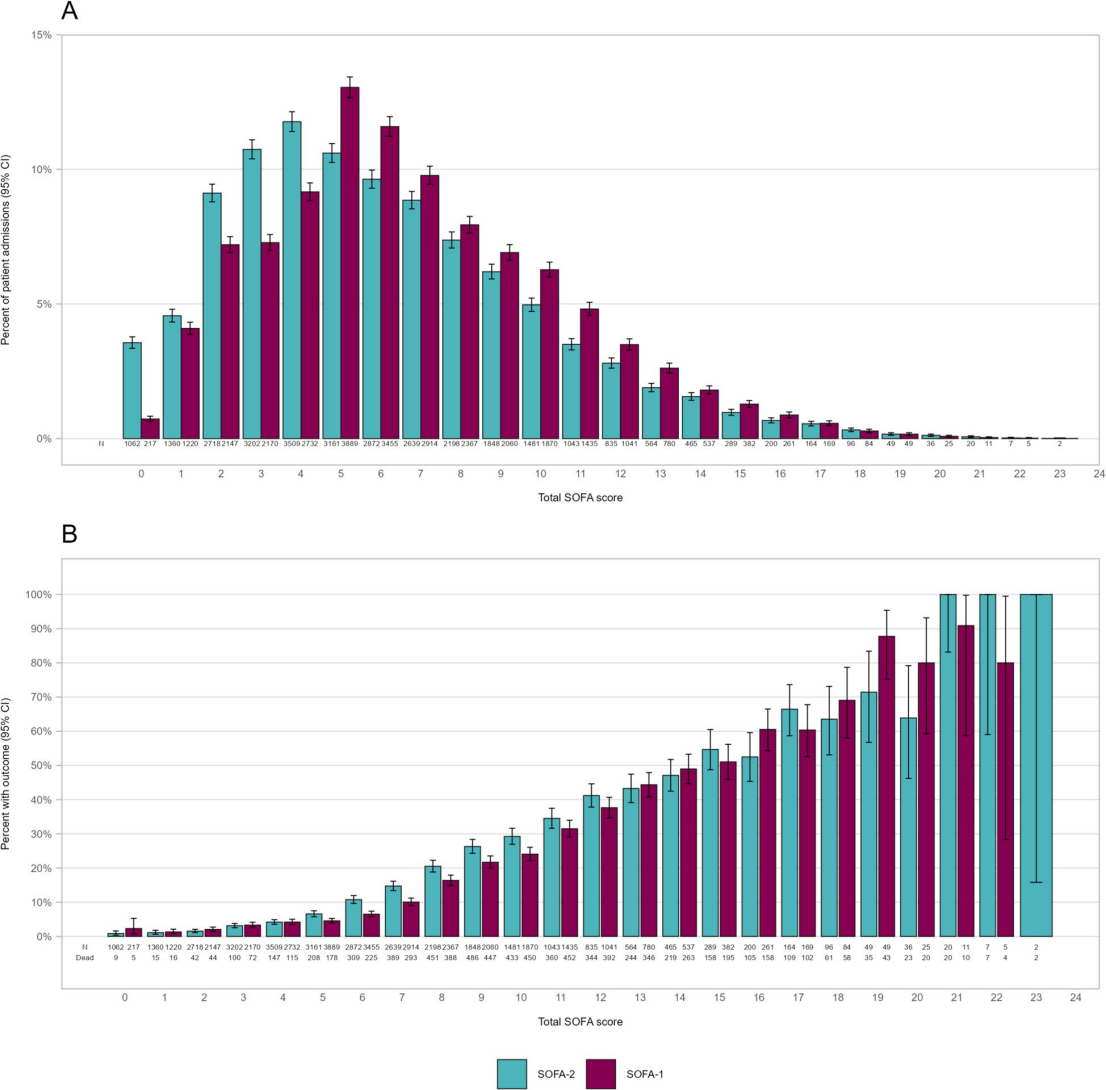
Distribution (**A**) and 30-day mortality (**B**) for total SOFA-2 and SOFA-1 at ICU day 1

**Fig. 2:**
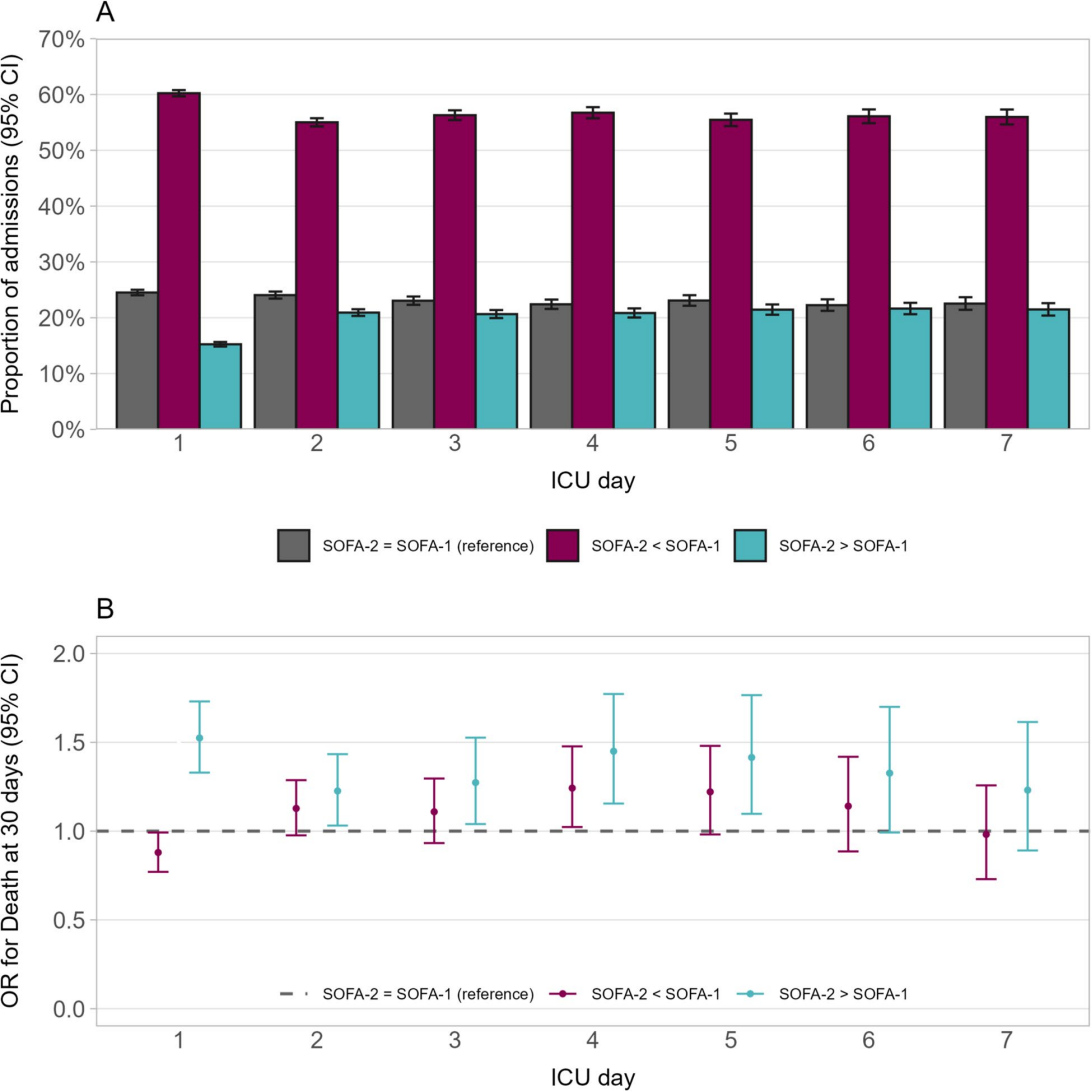
Reclassification between total SOFA-1 and SOFA-2 scores on ICU day 1–7 (**A**) and odds ratio (OR) for 30-day mortality according to reclassification status on ICU day 1–7 (**B**). The dashed line in panel B represents the reference category (equal SOFA-1 and SOFA-2 scores)

On day 1, the distribution of SOFA-2 (median [IQR] score 5 [3-8]) was shifted toward lower values compared with SOFA-1 (median [IQR] score 6 [4-9]) (Fig. 1A). Similar distributions were observed for ICU-day 2–7 (Supplementary Figure S2). Up to a score of approximately 15, SOFA-2 demonstrated a smoother and more consistently monotonic rise in 30-day mortality compared with SOFA-1. At higher scores, the pattern became less consistent, although the number of admissions contributing observations in this range was limited (Fig. 1B). Reclassification between SOFA-2 and SOFA-1 on day 1–7 is shown in Fig. 2A. On day 1, SOFA-2 and SOFA-1 scores were identical in 24.5% of admissions, SOFA-2 was higher than SOFA-1 in 15.3% and lower than SOFA-1 in 60.2%. Corresponding 30-day mortality was 14.3% when scores were identical, 20.3% when SOFA-2 was higher, and 12.8% when SOFA-2 was lower than SOFA-1. A similar pattern was seen on day 2–7. On logistic regression analysis, reclassification between SOFA-2 and SOFA-1 was associated with differences in mortality on day 1. Relative to patients with identical scores, SOFA-2 higher than SOFA-1 was associated with increased 30-day mortality (OR 1.52, 95% CI 1.33—1.73), whereas SOFA-2 lower than SOFA-1 was associated with decreased 30-day mortality (OR 0.88, 95% CI 0.77—0.99). On subsequent days, SOFA-2 scores higher than SOFA-1 was associated with increased risk of mortality up through day 5, but SOFA-2 scores lower than SOFA-1 did not show any association with 30-day mortality (Fig. 2B and Supplementary Table S1). The corresponding analysis for ICU mortality is shown in Supplementary Figure S3.

### Predictive validity of daily SOFA scores

On ICU-day 1, SOFA-2 had an AUROC of 0.81 (95% CI 0.80–0.81) for 30-day mortality, while SOFA-1 had an AUROC of 0.80 (95% CI 0.79–0.81), a statistically significant difference (p < 0.001). The optimism-corrected Brier score was 0.102 (95% CI 0.100 0.105) for SOFA-2 and 0.101 (95% CI 0.099—0.104) for SOFA-1. Calibration intercepts were 0.00 (95% CI –0.04—0.04) and 0.00 (95% CI –0.04—0.04) and slopes were 1.00 (95% CI 0.97—1.03) and 1.00 (95% CI 0.97—0.103) for SOFA-2 and SOFA-1, respectively. The NRI was 0.09 (95% CI 0.06—0.12), whereas there were no significant differences in IDI. For ICU mortality, AUROCs on day 1 were 0.84 (95% CI 0.83–0.84) for SOFA-2 and 0.83 (95% CI 0.82–0.84) for SOFA-1 (p < 0.001). Brier scores were 0.068 (95% CI 0.066—0.070) and 0.068 (95%CI 0.066—0.070), with calibration intercepts close to 0 and slopes close to 1 for both scores.

**Fig. 3:**
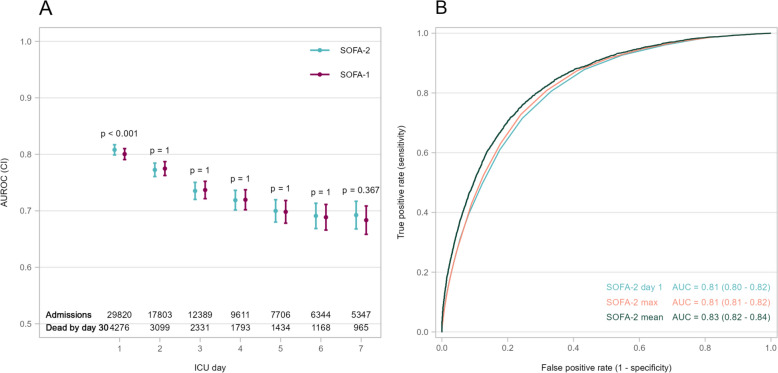
Area under the receiver operating characteristic (AUROC) curve of daily total SOFA-2 and SOFA-1 for 30-day mortality (**A**) and for maximum and mean SOFA-2 (**B**). P-values represents the comparison between SOFA-2 and SOFA-1 on each day and were Bonferroni-corrected for multiple comparisons

From day 2 through day 7, AUROCs for SOFA-2 and SOFA-1 gradually declined, while Brier scores slightly increased. No statistically significant differences in discrimination or calibration between the two scoring systems were observed beyond day 1 (Fig. 3A, Supplementary Tables S2a, S2b, Figure S4). On ICU days 2–4, NRI overall favoured SOFA-1 over SOFA-2, with no significant difference beyond that (supplementary table S3). We observed numerically lower AUROCs and higher Brier scores when using MICE (Supplementary Tables S4a, S4b) and in complete case analysis (Supplementary tables S5, S6). AUROCs for 30-day mortality were 0.83 (95% CI 0.82–0.84) for mean SOFA-2 and 0.82 (95% CI 0.81–0.83) for mean SOFA-1 (p < 0.001). AUROCs for maximum SOFA-2 and SOFA-1 were 0.81 (95% CI 0.81–0.82) and 0.80 (95% CI 0.80–0.81), respectively (p < 0.001). Corresponding AUROCs for ICU mortality were numerically higher (Fig. 3B, Supplementary Table S2a). On ICU day 1, the OR per point of SOFA-2 was 1.35 (95% CI 1.34- 1.36) for 30-day mortality, and 1.38 (95% CI 1.36—1.39) for ICU mortality.

### Predictive validity of SOFA-2 subscores

The predictive validity of domain-specific SOFA-2 subscores on 30-day mortality is shown in Supplementary Figure S5 and Table S7. On day 1, the brain subscore had the highest AUROC (0.73, 95% CI 0.72—0.74) and OR per 1-point increase (OR 1.60, 95% CI 1.57—1.64), while lowest predictive validity was seen in the liver and hemostasis domains. In the sensitivity analyses using mixed-effect models, the model including individual subscores had the best fit (lowest AIC), and the brain subscore remained the strongest within-admission predictor (conditional OR 1.32, 95% CI 1.18—1.47) (Supplementary table S8).

### Predictive validity of delta SOFA

Delta SOFA showed limited prognostic value on all days. Using either change relative to day 1 or change relative to the preceding day, all AUROCs for delta SOFA-2 and delta SOFA-1 were below 0.6 for 30-day mortality and between 0.56—0.62 for ICU mortality. (Supplementary Tables S9a and S9b).

### Subgroup analyses

**Fig. 4:**
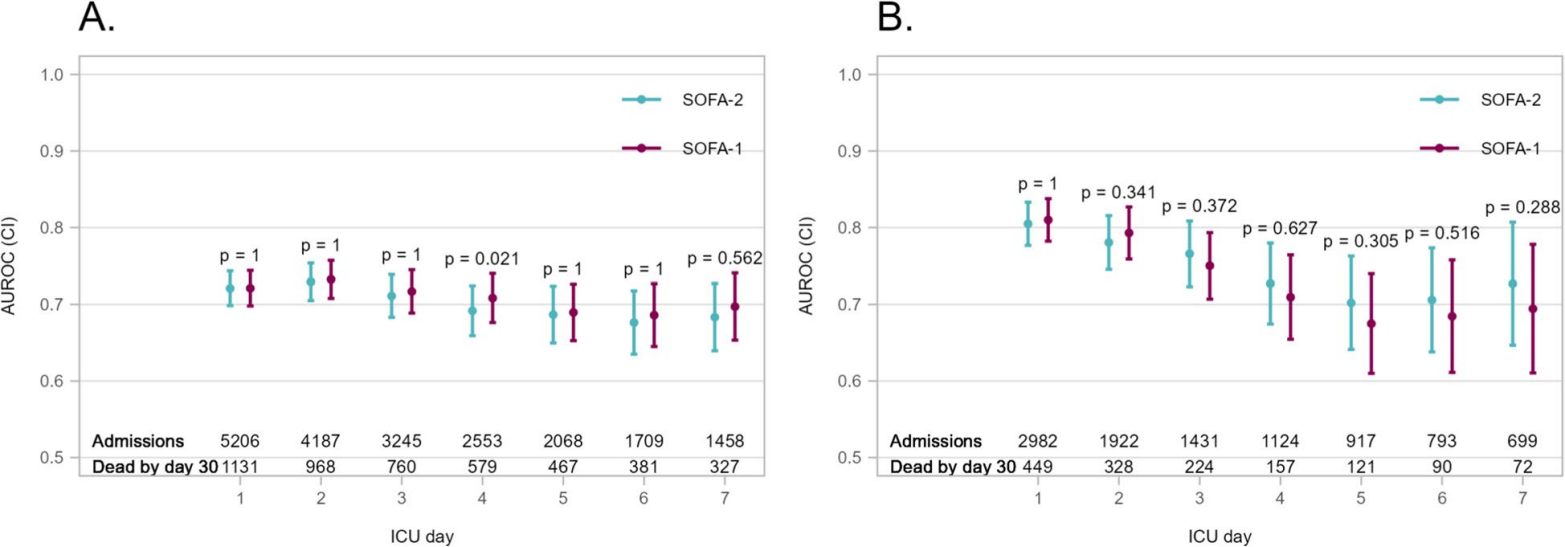
Area under the receiver operating characteristic (AUROC) curve of daily total SOFA-2 and SOFA-1 for 30-day mortality in patients with sepsis at admission (**A**) and in trauma patients (**B**)

Patients admitted following trauma were younger, more likely to be males, and had fewer comorbidities than patients with sepsis at admission (Supplementary Table S10). In the sepsis subgroup, AUROCs for SOFA-2 and SOFA-1 on 30-day mortality were consistently lower for ICU-day 1–4 than in the overall cohort while Brier scores were slightly higher. Additionally, SOFA-2 and SOFA-1 displayed similar predictive validity on each day (Fig. 4A, Supplementary Tables S11a, S11b). In the trauma subgroup, AUROCs for SOFA-2 were somewhat higher than in the overall cohort while Brier scores were lower than in the overall cohort without major differences between the scoring systems (Fig. 4B, Supplementary Tables S12a, S12b).

In the post-hoc analysis of patients with SOFA-1 ≥ 10 at admission (n = 3635, 30-day mortality 1626 [44.7%]), the median day 1 SOFA-2 score was 12 (IQR 10–14), compared with 13 (IQR 11–14) for SOFA-1. On ICU-day 1, AUROC for 30-day mortality was 0.62 (0.60—0.64) for SOFA-2 and 0.64 (0.62—0.66) for SOFA-1 (p = 0.023). SOFA-1 also had a lower Brier score in this cohort (supplementary tables S13, S14a, S14b and Figure S6).

### Missing data

Missing data were most prevalent in liver (27.7%) and coagulation/hemostasis (14.2%) on day 1, while respiratory and renal domains were nearly complete (> 99%). Missingness decreased on subsequent ICU days (Supplementary Tables S15a–b). Sensitivity analyses using multiple imputation resulted in worse discrimination than the primary approach of assuming normal when missing and using last-observation-carried-forward (LOCF) for both scoring systems, with a greater decrease for SOFA-2 than SOFA-1. The majority of LOCF imputations were score 0 (SOFA-1 53.7% and SOFA-2 52.7%). The agreement between SOFA-1 and SOFA-2 and the direction of all primary findings were unchanged.

### Organ failure agreement

Agreement between SOFA-2 and SOFA-1 in classifying organ failure (subscore ≥ 2) on day 1 was highest for hemostasis (> 99.9%), cardiovascular (99.4%), and brain (98.8%), and lowest for the respiratory domain (78.4%), reflecting the different PaO₂/FiO₂ thresholds (Supplementary Table S16).

## Discussion

### Key findings

In this observational study of nearly 30,000 ICU admissions, we compared the predictive performance of SOFA-2 with the original SOFA-1 during the first ICU week using 30-day mortality as the primary endpoint. On ICU day 1, SOFA-2 overall demonstrated a small but statistically significant improvement in discrimination, but that did not translate into a clinically meaningful improvement in overall predictive accuracy as measured by the Brier score. Both scoring systems showed a decreasing and similar predictive performance across the subsequent ICU days without any significant differences in discrimination or calibration. Overall, the mean SOFA-2 across the entire ICU stay was the strongest single predictor of 30-day mortality.

When examining subgroups, SOFA-2 demonstrated higher predictive accuracy in patients admitted after trauma, a younger, less comorbid cohort, and lower accuracy in patients admitted with sepsis, who were older and carried a greater comorbidity burden. In the post-hoc analysis of patients admitted with SOFA-1 ≥ 10, SOFA-2 had a lower AUROC than SOFA-1, but the overall discrimination of both scoring systems was low. Of the individual subscores, the brain subscore demonstrated the strongest association with 30-day mortality both in terms of discrimination and OR per point.

### Relationship with previous studies

Our results align with, but do not fully replicate, the findings from the original SOFA-2 validation study [3]. Compared with that cohort, our patients were younger on average (60 vs 65 years), less often female (35% vs 41%), and presented with greater initial organ dysfunction, reflected in a higher median day-1 SOFA-2 score (5 vs 3). Despite a substantially larger proportion of reclassifications between SOFA-1 and SOFA-2 in our study (75% vs 50%), the predictive validity for ICU mortality in our cohort was consistent with that reported previously. This concordance held across all commonly used SOFA measures, including day-1, mean, maximum, and delta scores. Day-1 SOFA-2 showed a statistically detectable but small increase in discriminative performance compared to SOFA-1, which is unlikely to change clinical practice.

Our study extends the original work by demonstrating that both day-1 and mean SOFA-2 scores also show robust discrimination for 30-day mortality, an outcome not evaluated in the initial validation. We further corroborate that, among the six organ-specific subscores, the brain score contributes most strongly to mortality prediction, an observation also highlighted in the original analysis. Using cluster-robust logistic regression and generalized mixed-effect models, we additionally show that the brain subscore is the single most informative SOFA-2 component and appears to contribute disproportionately to the association between SOFA-2 score and mortality, reinforcing its central role in risk stratification.

Although SOFA-2 reclassified 75–79% of admissions on days 1–7, NRI tended to be small and varied in direction across days, and IDI showed no meaningful difference. The recalibration may relabel a patient’s absolute score without substantially altering their position in the prognostic risk ranking. Fixed SOFA-1 cutoffs (including the ≥ 2-point increase in Sepsis-3) might not transfer directly to SOFA-2, and for severity ranking the two scores seem largely interchangeable over the first ICU week in our cohort.

The weakening performance of serial SOFA measurements over time suggests that early physiological derangements carry prognostic weight on admission but become less informative later in the ICU course. By the end of the first week, many patients who remain in the ICU represent a fundamentally different population than the ones captured by day-1 acute physiology. In these longer-stay patients, survival may be less a function of the initial acute insult and more influenced by factors such as underlying comorbidity, frailty, and complications arising during the ICU stay. This trajectory mirrors the population-level transition described by Iwashyna and colleagues [15], who showed that acute physiological severity and admission diagnosis, which strongly predicted mortality on day 1, progressively lost discriminative ability over time and no longer outperformed antecedent patient characteristics after approximately 10 days. However, the observed decline in predictive performance may also reflect survivor bias and competing risks, as later SOFA measurements are only available in patients who remained alive and in the ICU long enough to be assessed, while death and discharge both remove patients from subsequent observations.

Our subgroup results are consistent with this broader interpretation of how SOFA functions as a prognostic tool. In our trauma cohort, SOFA achieved substantially higher discrimination for 30-day mortality (AUROC 0.80) than in the sepsis cohort (AUROC 0.72). These values align with previous trauma-studies reporting AUROCs in the range of 0.81–0.94 [16, 17] and with sepsis-studies reporting lower values around 0.67–0.69 [18–20]. The findings should be interpreted cautiously as the subgroup analyses were exploratory. The divergence may reflect underlying differences in case mix and sample size rather than limitations of the score itself.

### Implications of study findings

Our finding that SOFA-2 has better discrimination than SOFA-1 on ICU day 1 for 30-day mortality supports the use of SOFA-2 as a more informative scoring system for multiorgan failure, particularly in the early phase of ICU care. The superior performance of mean SOFA-2 compared to maximum SOFA-2 implies that persistent multiorgan failure, rather than transient peaks, may be a key driver of mortality and reinforces the importance of serial reassessment of organ dysfunction, consistent with earlier findings that hourly average SOFA-1 is a strong predictor of mortality [21].

Our finding that SOFA-2 performance differ between sepsis and trauma patients implies that age and baseline health status, in addition to the triggering cause of multiorgan failure, may modify the relationship between SOFA-2 and mortality. These observed differences warrant external validation and further work to identify underlying mechanisms.

The observation that the brain subscore is the strongest SOFA-2 component for predicting mortality underscores the importance of systematic neurologic assessment and supports the practice of carrying forward pre-sedation GCS values in EHR-based SOFA-2 calculations, with preserved prognostic information under the updated brain scoring rules. Finally, we have shown that in our setting, treating unmeasured SOFA-2 components as normal and carrying forward last observed values yields better prognostic performance than multiple imputation, supporting this pragmatic approach in retrospective studies.

### Strengths and limitations

Our study has strengths. To the best of our knowledge, it is the first to validate daily SOFA-2 against 30-day mortality thereby closing an important gap in the literature. Importantly, our study population is entirely independent from the original SOFA-2 development and validation cohorts, providing an external validation in a different health system and organisational context. Finally, we used a carefully curated high-resolution database, which increases the internal validity of our findings.

Our study has limitations. All participating ICUs were located in the capital region of a single, high-income country, which may limit generalizability to other regions, resource-limited settings, and systems with different admission and discharge practices. We lacked information on treatment limitations, which could influence both SOFA scoring and subsequent outcomes. In addition, pre-admission SOFA was unavailable, preventing assessment of the contribution of chronic organ dysfunction to SOFA-2 performance. Furthermore, the complete SOFA-2 algorithm has not been validated against manual chart review, and agreement remains unquantified. While most components were validated during our SOFA-1 algorithm development, the SOFA-2-specific components (delirium-related medication, ECMO, and RRT) were not. The cohort included elective admissions, resulting in relatively low median SOFA scores, a relatively high frequency of missing data particularly in the liver component, and short ICU length of stay, which should be considered when comparing these findings to cohorts with a higher proportion of severely ill patients. Finally, we applied LOCF^3,14^ on days 2–7, despite its known potential to dampen day-to-day variation. However, sensitivity analyses (complete-case and multiple imputation) reproduced the same pattern, (Supplementary Tables S4a-b, S5 and S6). Yet, residual LOCF bias from temporal smoothing cannot be excluded.

## Conclusion

In our cohort, SOFA-2 offered a small but measurable improvement in discrimination for 30-day mortality at the start of ICU care compared with SOFA-1. This advantage did not persist beyond the first day, as both scores showed declining and largely similar predictive validity over the remaining first week. Performance differed across clinical subgroups with distinct age and comorbidity patterns, indicating that the usefulness of either scoring system depends on the underlying case mix.

## Supplementary Information

Additional file 1.

## Data Availability

The data that support the findings of this study are not openly available due to reasons of sensitivity and are available from the corresponding author upon reasonable request. Data are located in controlled access data storage at Karolinska Institutet. Example from: (10.1186/s12910-022-00758-z).
