# Supplementary material for: Predictive validity of daily sequential organ failure assessment (SOFA)-2 score for 30-day mortality
Source: Crit Care. 2026 Jun 1;30:280. doi: 10.1186/s13054-026-06093-8 (PMC13224550; doi:10.1186/s13054-026-06093-8)
Supplement: Supplementary file 1 — Additional file 1. [file 13054_2026_6093_MOESM1_ESM.docx]

**Supplementary Material**

Helleberg J, Sundelin A, Soltani N, Rooyackers O, Mårtensson J. Predictive validity of daily sequential organ failure assessment (SOFA)-2 score for 30-day mortality.

Table of Contents

[Figure S1. Patient selection. 3](#_Toc228447620)

[Figure S2. Distribution for total SOFA-2 and SOFA-1 at ICU day 2-7 4](#_Toc228447621)

[Table S1. Odds ratio for 30-day mortality based on reclassification status on day 1-7. Reference group is no reclassification (SOFA-2 = SOFA-1). 5](#_Toc228447622)

[Figure S3. OR for ICU mortality according to reclassification status. The dashed line represents the reference category (equal SOFA-1 and SOFA-2 scores). 6](#_Toc228447623)

[Table S2a. AUROC of daily total SOFA-2 and SOFA-1 for 30-day and ICU mortality using LOCF imputation 7](#_Toc228447624)

[Table S2b. Calibration metrics for SOFA-2 and SOFA-1 for 30-day and ICU mortality using LOCF imputation 7](#_Toc228447625)

[Table S3. Net reclassification improvement (NRI) and integrated discrimination improvement (IDI) for SOFA-2 versus SOFA-1 in predicting 30-day mortality at each ICU day. Full cohort, LOCF imputation. Positive values indicate SOFA-2 classifies better than SOFA-1. Category-free NRI with 1000 bootstrap iterations. IDI separated between those with the outcome and those without. 9](#_Toc228447626)

[Table S4a. AUROC of daily total SOFA-2 and SOFA-1 for 30-day and ICU mortality using MICE imputation 10](#_Toc228447627)

[Table S4b. Calibration metrics for SOFA-2 and SOFA-1 for 30-day and ICU mortality using MICE imputation 10](#_Toc228447628)

[Table S5. Complete case analysis. Discrimination for SOFA-2 and SOFA-1 at each ICU day. 12](#_Toc228447629)

[Figure S4: Calibration plot for the univariate logistic regression models with SOFA-2 or SOFA-1 as predictors and 30-day mortality as outcome. Dots: Predicted probability and observed outcome (0 = alive at 30 days, 1 = dead at 30 days) ; Lines: Calibration line with optimism correction; Shaded areas and dotted lines : 95% confidence intervals for calibration line from bootstrap resampling. 13](#_Toc228447630)

[Figure S5. Daily AUROC per SOFA-2 subscore (A) and adjusted OR per 1-unit increase in subscore (B) on 30-day mortality. OR with cluster adjusted confidence intervals and Bonferroni correction. Abbreviations: AUROC area under the receiver operating characteristic curve. OR odds ratio. ICU intensive care unit. CI confidence interval. 14](#_Toc228447631)

[Table S6. Complete case analysis. Calibration data for SOFA-2 and SOFA-1 at each ICU day. 14](#_Toc228447632)

[Table S7. ROC-areas per SOFA-2 component for 30-day mortality 15](#_Toc228447633)

[Table S8. Conditional OR per point per domain from generalized mixed-effect model with binomial link and patient-level random intercept. 15](#_Toc228447634)

[Table S9a. AUROC of delta SOFA-2 and delta SOFA-1 for 30-day mortality 16](#_Toc228447635)

[Table S9b. AUROC of delta SOFA-2 and delta SOFA-1 for ICU mortality 16](#_Toc228447636)

[Table S10. Characteristics of ICU patients admitted due to trauma or sepsis 17](#_Toc228447637)

[Table S11a. AUROC for SOFA-2 and SOFA-1 for 30-day mortality in patients with sepsis. 18](#_Toc228447638)

[Table S11b. Calibration metrics for SOFA-2 and SOFA-1 for 30-day mortality in patients with sepsis. 18](#_Toc228447639)

[Table S12a. AUROC for SOFA-2 and SOFA-1 for 30-day mortality in trauma patients. 19](#_Toc228447640)

[Table S12b. Calibration metrics for SOFA-2 and SOFA-1 for 30-day mortality in patients with trauma. 19](#_Toc228447641)

[Table S13. Baseline characteristics of ICU admissions with SOFA-1 score ≥10 on ICU arrival. 20](#_Toc228447642)

[Table S14a. AUROC for SOFA-2 and SOFA-1 for 30-day mortality in patients with SOFA-1 ≥10 at admission 20](#_Toc228447643)

[Table S14b. Calibration metrics for SOFA-2 and SOFA-1 for 30-day mortality in patients with SOFA-1 ≥10 at admission 21](#_Toc228447644)

[Figure S6. AUROCs with 95% CI for patients admitted with SOFA-1 score ≥10 (A) 22](#_Toc228447645)

[Table S15a. Missing per domain SOFA-1. 22](#_Toc228447646)

[Table S15b. Missing per domain SOFA-2. 23](#_Toc228447647)

[Table S16. Agreement of failure (score ≥2) per domain between SOFA-2 and SOFA-1 on ICU day 1. 23](#_Toc228447648)


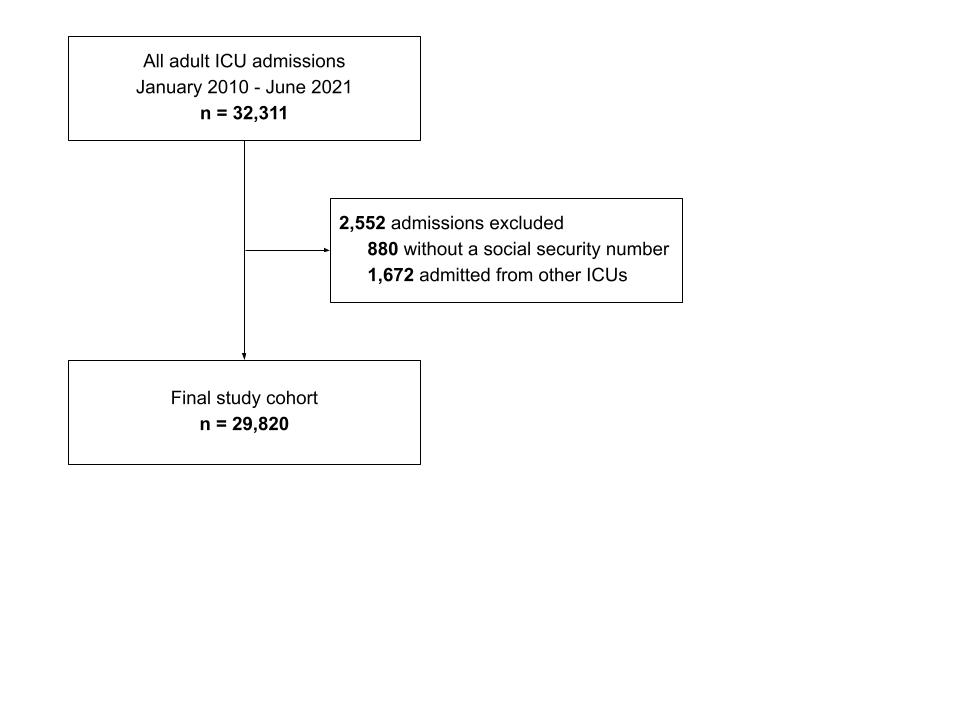


## **Figure S1**. Patient selection.


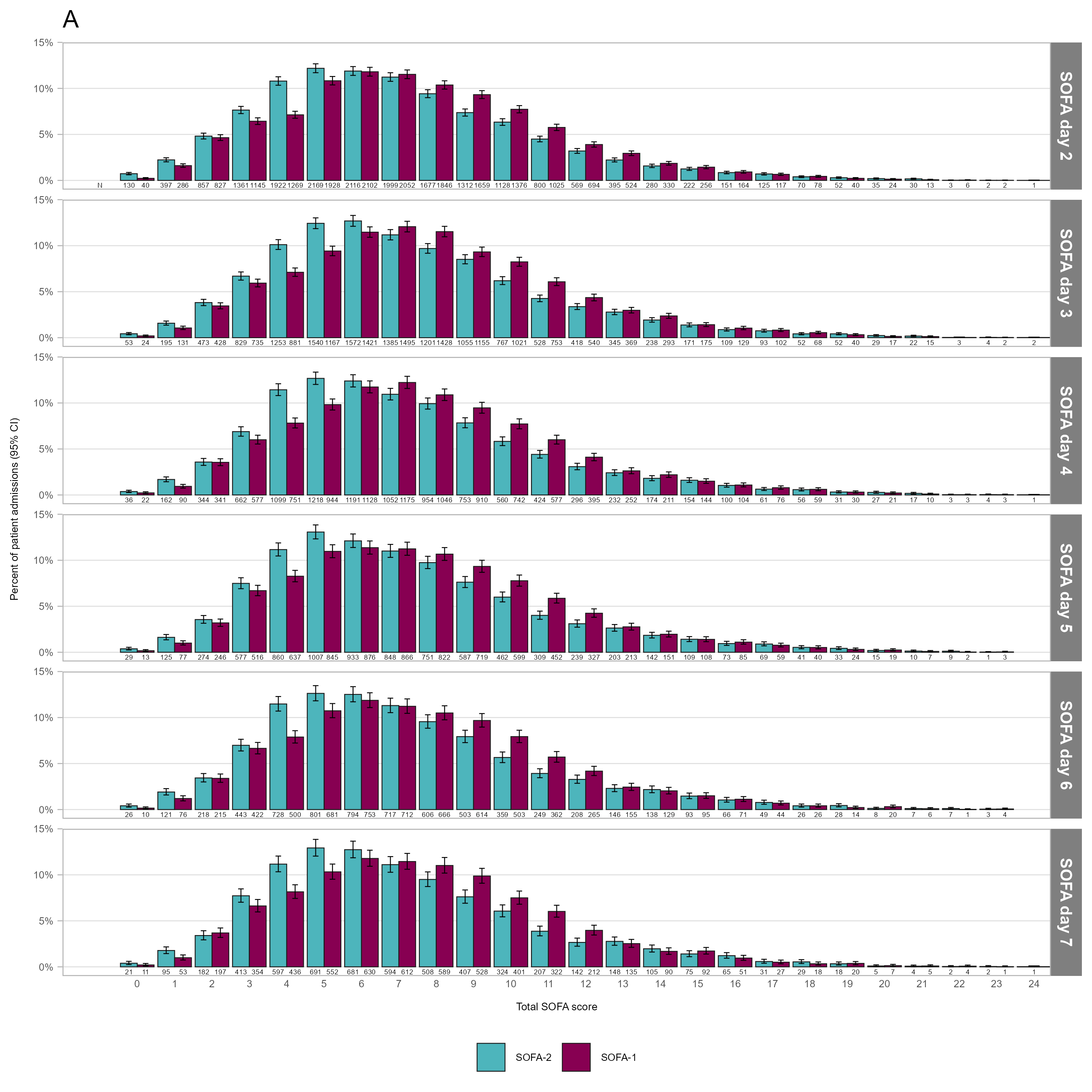


## **Figure S2**. Distribution for total SOFA-2 and SOFA-1 at ICU day 2-7

## Table **S1.** Odds ratio for 30-day mortality based on reclassification status on day 1-7. Reference group is no reclassification (SOFA-2 = SOFA-1).

|  | **SOFA-2 > SOFA-1** | | **SOFA-2 < SOFA-1** | |
| --- | --- | --- | --- | --- |
| **ICU day** | **OR (95% CI)** | **p-value** | **OR (95% CI)** | **p-value** |
| 1 | 1.52 (1.33 - 1.73) | p < 0.001 | 0.88 (0.77 - 0.99) | p = 0.021 |
| 2 | 1.23 (1.03 - 1.43) | p = 0.009 | 1.13 (0.98 - 1.29) | p = 0.227 |
| 3 | 1.27 (1.04 - 1.53) | p = 0.008 | 1.11 (0.93 - 1.30) | p = 1.000 |
| 4 | 1.45 (1.16 - 1.77) | p < 0.001 | 1.24 (1.02 - 1.48) | p = 0.024 |
| 5 | 1.41 (1.10 - 1.77) | p = 0.001 | 1.22 (0.98 - 1.48) | p = 0.129 |
| 6 | 1.33 (0.99 - 1.70) | p = 0.063 | 1.14 (0.89 - 1.42) | p = 1.000 |
| 7 | 1.23 (0.89 - 1.61) | p = 0.759 | 0.98 (0.73 - 1.26) | p = 1.000 |


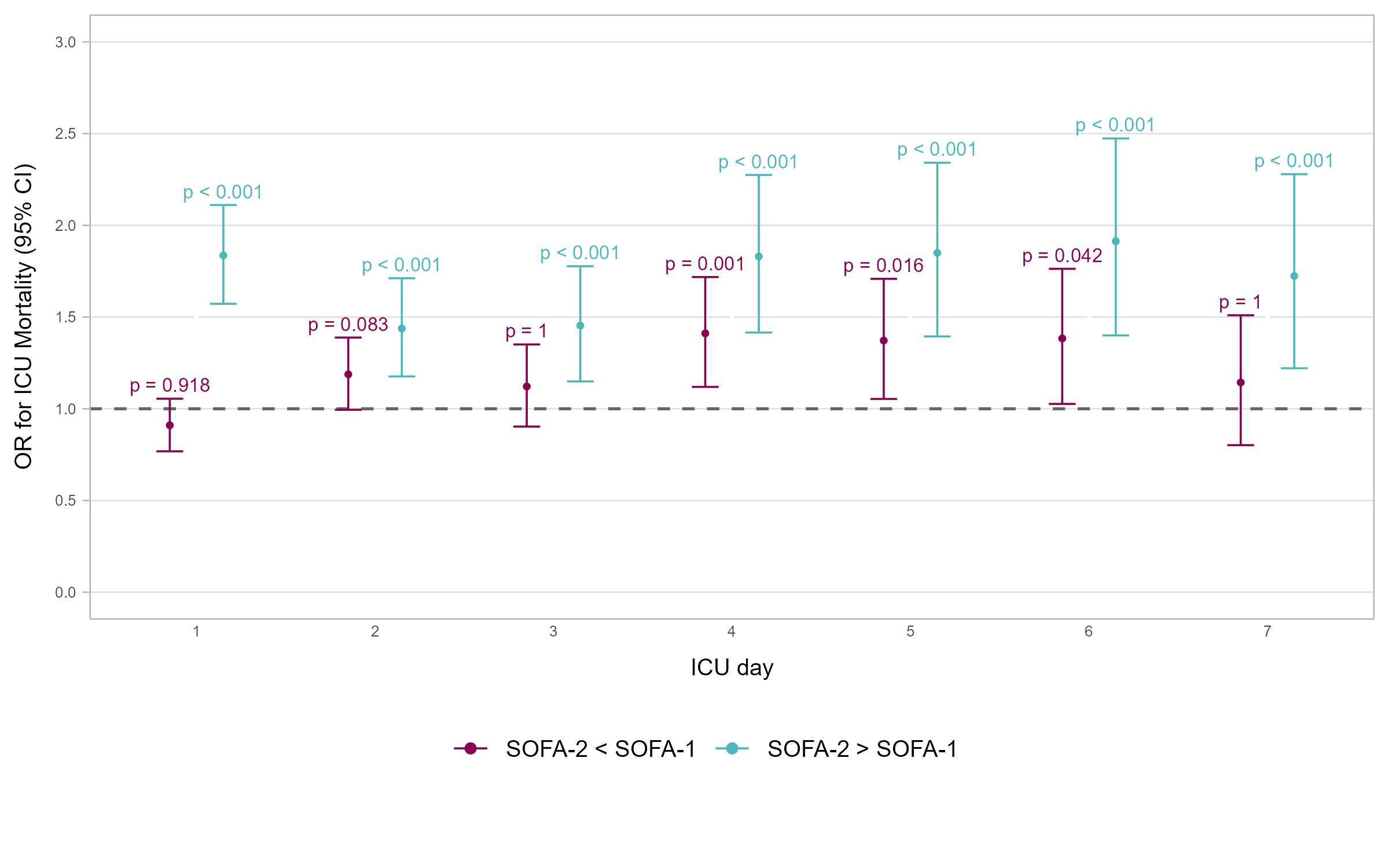


## **Figure S3**. OR for ICU mortality according to reclassification status. The dashed line represents the reference category (equal SOFA-1 and SOFA-2 scores).

## **Table S2a**. AUROC of daily total SOFA-2 and SOFA-1 for 30-day and ICU mortality using LOCF imputation

| 30-day mortality | | | | |
| --- | --- | --- | --- | --- |
| **ICU day** | **No. of deaths/**  **No. of admissions** | **SOFA-2 AUROC**  **(95% CI)** | **SOFA-1 AUROC**  **(95% CI)** | **p-value** |
| 1 | 4276/29820 | 0.81 (0.80 - 0.81) | 0.80 (0.79 - 0.81) | p < 0.001 |
| 2 | 3099/17803 | 0.77 (0.76 - 0.78) | 0.77 (0.77 - 0.78) | p = 1.000 |
| 3 | 2331/12389 | 0.74 (0.72 - 0.75) | 0.74 (0.73 - 0.75) | p = 1.000 |
| 4 | 1793/9611 | 0.72 (0.71 - 0.73) | 0.72 (0.71 - 0.73) | p = 1.000 |
| 5 | 1434/7706 | 0.70 (0.69 - 0.71) | 0.70 (0.68 - 0.71) | p = 1.000 |
| 6 | 1168/6344 | 0.69 (0.67 - 0.71) | 0.69 (0.67 - 0.71) | p = 1.000 |
| 7 | 965/5347 | 0.69 (0.67 - 0.71) | 0.68 (0.67 - 0.70) | p = 0.366 |
| Mean | 4276/29820 | 0.83 (0.82 - 0.84) | 0.82 (0.81 - 0.83) | p < 0.001 |
| Max | 4276/29820 | 0.81 (0.81 - 0.82) | 0.80 (0.80 - 0.81) | p < 0.001 |
| ICU mortality | | | | |
| **ICU day** | **No. of deaths/**  **No. of admissions** | **SOFA-2 AUROC**  **(95% CI)** | **SOFA-1 AUROC**  **(95% CI)** | **p-value** |
| 1 | 2643/29820 | 0.84 (0.83 - 0.84) | 0.83 (0.82 - 0.84) | p < 0.001 |
| 2 | 1934/17803 | 0.80 (0.79 - 0.81) | 0.81 (0.80 - 0.82) | p = 1.000 |
| 3 | 1460/12389 | 0.77 (0.75 - 0.78) | 0.77 (0.75 - 0.78) | p = 1.000 |
| 4 | 1129/9611 | 0.74 (0.73 - 0.76) | 0.74 (0.73 - 0.76) | p = 1.000 |
| 5 | 909/7706 | 0.73 (0.72 - 0.75) | 0.73 (0.71 - 0.74) | p = 0.875 |
| 6 | 750/6344 | 0.73 (0.71 - 0.75) | 0.72 (0.70 - 0.74) | p = 1.000 |
| 7 | 640/5347 | 0.73 (0.70 - 0.75) | 0.71 (0.69 - 0.73) | p = 0.157 |
| Mean | 2643/29820 | 0.87 (0.86 - 0.87) | 0.86 (0.85 - 0.86) | p < 0.001 |
| Max | 2643/29820 | 0.86 (0.85 - 0.86) | 0.84 (0.84 - 0.85) | p < 0.001 |
| AUROC, area under the receiver operating characteristic curve; LOCF, last-observation-carried-forward; Mean SOFA throughout ICU stay; Max: Maximum SOFA throughout ICU stay  Daily 95% CIs and p-values were Bonferroni-corrected for multiple comparisons. | | | | |

## **Table S2b**. Calibration metrics for SOFA-2 and SOFA-1 for 30-day and ICU mortality using LOCF imputation

| 30-day mortality | | | |  |  |  |
| --- | --- | --- | --- | --- | --- | --- |
| **ICU day** | **SOFA-2**  **Brier (95% CI)** | **SOFA-1**  **Brier (95% CI)** | **SOFA-2**  **intercept (95% CI)** | **SOFA-1**  **intercept (95% CI)** | **SOFA-2**  **slope**  **(95% CI)** | **SOFA-1**  **slope**  **(95% CI)** |
| 1 | 0.102 (0.100 - 0.105) | 0.101 (0.099 - 0.104) | 0.000 (-0.036 - 0.036) | 0.000 (-0.035 - 0.036) | 1.000 (0.968 - 1.032) | 1.000 (0.968 - 1.034) |
| 2 | 0.123 (0.120 - 0.126) | 0.121 (0.117 - 0.124) | 0.001 (-0.039 - 0.044) | 0.001 (-0.039 - 0.045) | 1.000 (0.958 - 1.044) | 1.000 (0.958 - 1.044) |
| 3 | 0.135 (0.131 - 0.139) | 0.134 (0.130 - 0.138) | 0.000 (-0.045 - 0.048) | 0.000 (-0.046 - 0.051) | 1.000 (0.944 - 1.054) | 1.000 (0.941 - 1.057) |
| 4 | 0.138 (0.133 - 0.143) | 0.137 (0.132 - 0.141) | 0.000 (-0.053 - 0.055) | 0.000 (-0.056 - 0.056) | 1.002 (0.938 - 1.074) | 1.002 (0.939 - 1.074) |
| 5 | 0.140 (0.135 - 0.145) | 0.139 (0.134 - 0.144) | 0.000 (-0.058 - 0.059) | 0.000 (-0.059 - 0.061) | 1.001 (0.917 - 1.089) | 1.001 (0.920 - 1.087) |
| 6 | 0.139 (0.133 - 0.145) | 0.139 (0.133 - 0.144) | -0.001 (-0.068 - 0.069) | -0.001 (-0.069 - 0.065) | 1.003 (0.915 - 1.099) | 1.002 (0.916 - 1.101) |
| 7 | 0.137 (0.130 - 0.142) | 0.136 (0.130 - 0.142) | 0.000 (-0.070 - 0.074) | 0.000 (-0.069 - 0.073) | 1.002 (0.909 - 1.108) | 1.003 (0.910 - 1.115) |
| ICU mortality | | | |  |  |  |
| **ICU day** | **SOFA-2**  **Brier (95% CI)** | **SOFA-1**  **Brier (95% CI)** | **SOFA-2**  **intercept (95% CI)** | **SOFA-1**  **intercept (95% CI)** | **SOFA-2**  **slope**  **(95% CI)** | **SOFA-1**  **slope**  **(95% CI)** |
| 1 | 0.068 (0.066 - 0.070) | 0.068 (0.066 - 0.070) | -0.001 (-0.042 - 0.046) | 0.000 (-0.042 - 0.045) | 1.000 (0.967 - 1.035) | 1.000 (0.967 - 1.039) |
| 2 | 0.083 (0.080 - 0.086) | 0.082 (0.079 - 0.085) | 0.001 (-0.053 - 0.051) | 0.001 (-0.052 - 0.051) | 1.001 (0.956 - 1.046) | 1.001 (0.956 - 1.045) |
| 3 | 0.092 (0.088 - 0.096) | 0.092 (0.088 - 0.095) | -0.001 (-0.065 - 0.060) | -0.001 (-0.066 - 0.059) | 1.000 (0.949 - 1.060) | 1.000 (0.943 - 1.060) |
| 4 | 0.094 (0.090 - 0.098) | 0.094 (0.089 - 0.098) | 0.000 (-0.063 - 0.066) | 0.000 (-0.064 - 0.066) | 1.001 (0.931 - 1.073) | 1.002 (0.933 - 1.076) |
| 5 | 0.096 (0.090 - 0.100) | 0.096 (0.090 - 0.100) | 0.002 (-0.069 - 0.076) | 0.002 (-0.069 - 0.075) | 1.001 (0.930 - 1.087) | 1.001 (0.927 - 1.080) |
| 6 | 0.096 (0.090 - 0.101) | 0.096 (0.090 - 0.101) | -0.001 (-0.079 - 0.079) | -0.001 (-0.079 - 0.076) | 0.998 (0.915 - 1.087) | 0.999 (0.914 - 1.104) |
| 7 | 0.096 (0.090 - 0.102) | 0.097 (0.090 - 0.102) | 0.002 (-0.078 - 0.092) | 0.003 (-0.081 - 0.094) | 1.005 (0.914 - 1.112) | 1.006 (0.910 - 1.126) |
| Brier, intercept and slope optimism corrected and 95% CIs computed using bootstrap resampling. | | | | | | |

| **ICU day** | **n** | **NRI (95% CI)** | **IDI events (95% CI)** | **IDI non-events (95% CI)** |
| --- | --- | --- | --- | --- |
| 1 | 29,820 | 0.088 (0.055 - 0.119) | -0.001 (-0.004 - 0.002) | 0 (-0.001 - 0) |
| 2 | 17,803 | -0.162 (-0.2 - -0.121) | -0.011 (-0.014 - -0.007) | -0.002 (-0.003 - -0.001) |
| 3 | 12,389 | -0.057 (-0.102 - -0.012) | -0.004 (-0.007 - -0.001) | -0.001 (-0.002 - 0) |
| 4 | 9,611 | -0.062 (-0.111 - -0.011) | -0.004 (-0.008 - -0.001) | -0.001 (-0.002 - 0) |
| 5 | 7,706 | -0.043 (-0.095 - 0.016) | -0.003 (-0.007 - 0) | -0.001 (-0.002 - 0) |
| 6 | 6,344 | -0.016 (-0.078 - 0.046) | -0.003 (-0.007 - 0.001) | -0.001 (-0.002 - 0.001) |
| 7 | 5,347 | 0.047 (-0.019 - 0.112) | 0 (-0.004 - 0.004) | 0 (-0.001 - 0.002) |

## **Table S3.** Net reclassification improvement (NRI) and integrated discrimination improvement (IDI) for SOFA-2 versus SOFA-1 in predicting 30-day mortality at each ICU day. Full cohort, LOCF imputation. Positive values indicate SOFA-2 classifies better than SOFA-1. Category-free NRI with 1000 bootstrap iterations. IDI separated between those with the outcome and those without.

## **Table** S4a. AUROC of daily total SOFA-2 and SOFA-1 for 30-day and ICU mortality using MICE imputation

| 30-day mortality | | | | |
| --- | --- | --- | --- | --- |
| **ICU day** | **No. of deaths/**  **No. of admissions** | **SOFA-2 AUROC**  **(95% CI)** | **SOFA-1 AUROC**  **(95% CI)** | **p-value** |
| 1 | 4276/29820 | 0.78 (0.78 - 0.79) | 0.79 (0.79 - 0.80) | p < 0.001 |
| 2 | 3099/17803 | 0.76 (0.75 - 0.76) | 0.77 (0.76 - 0.78) | p < 0.001 |
| 3 | 2331/12389 | 0.72 (0.71 - 0.73) | 0.73 (0.71 - 0.74) | p = 0.105 |
| 4 | 1793/9611 | 0.70 (0.69 - 0.72) | 0.71 (0.70 - 0.72) | p = 0.684 |
| 5 | 1434/7706 | 0.69 (0.68 - 0.71) | 0.69 (0.68 - 0.71) | p = 1.000 |
| 6 | 1168/6344 | 0.69 (0.67 - 0.71) | 0.69 (0.67 - 0.71) | p = 1.000 |
| 7 | 965/5347 | 0.68 (0.66 - 0.70) | 0.68 (0.66 - 0.70) | p = 1.000 |
| ICU mortality | | | | |
| **ICU day** | **No. of deaths/**  **No. of admissions** | **SOFA-2 AUROC**  **(95% CI)** | **SOFA-1 AUROC**  **(95% CI)** | **p-value** |
| 1 | 2643/29820 | 0.82 (0.81 - 0.83) | 0.83 (0.82 - 0.83) | p = 0.002 |
| 2 | 1934/17803 | 0.79 (0.78 - 0.80) | 0.80 (0.79 - 0.81) | p = 0.012 |
| 3 | 1460/12389 | 0.75 (0.74 - 0.76) | 0.76 (0.74 - 0.77) | p = 0.269 |
| 4 | 1129/9611 | 0.73 (0.71 - 0.74) | 0.74 (0.72 - 0.75) | p = 0.689 |
| 5 | 909/7706 | 0.72 (0.71 - 0.74) | 0.72 (0.71 - 0.74) | p = 1.000 |
| 6 | 750/6344 | 0.73 (0.71 - 0.75) | 0.73 (0.71 - 0.75) | p = 1.000 |
| 7 | 640/5347 | 0.71 (0.69 - 0.73) | 0.71 (0.69 - 0.74) | p = 1.000 |
| AUROC, area under the receiver operating characteristic curve; MICE, multiple imputation with chained equations  Daily 95% CIs and p-values were Bonferroni-corrected for multiple comparisons | | | | |

## Table **S4b.** Calibration metrics for SOFA-2 and SOFA-1 for 30-day and ICU mortality using MICE imputation

| 30-day mortality | | | |  |  |  |
| --- | --- | --- | --- | --- | --- | --- |
| **ICU day** | **SOFA-2**  **Brier (95% CI)** | **SOFA-1**  **Brier (95% CI)** | **SOFA-2**  **intercept (95% CI)** | **SOFA-1**  **intercept (95% CI)** | **SOFA-2**  **slope**  **(95% CI)** | **SOFA-1**  **slope**  **(95% CI)** |
| 1 | 0.105 (0.102 - 0.107) | 0.102 (0.100 - 0.105) | 0.000 (-0.036 - 0.037) | 0.000 (-0.034 - 0.039) | 1.000 (0.966 - 1.032) | 1.000 (0.967 - 1.033) |
| 2 | 0.125 (0.122 - 0.128) | 0.122 (0.119 - 0.125) | 0.000 (-0.041 - 0.042) | 0.000 (-0.042 - 0.042) | 1.000 (0.959 - 1.047) | 1.000 (0.960 - 1.043) |
| 3 | 0.138 (0.134 - 0.141) | 0.136 (0.132 - 0.140) | 0.000 (-0.048 - 0.045) | 0.000 (-0.049 - 0.045) | 0.999 (0.937 - 1.057) | 1.000 (0.941 - 1.060) |
| 4 | 0.139 (0.134 - 0.143) | 0.138 (0.133 - 0.143) | -0.001 (-0.053 - 0.053) | -0.001 (-0.055 - 0.053) | 1.001 (0.937 - 1.080) | 1.001 (0.937 - 1.078) |
| 5 | 0.140 (0.135 - 0.145) | 0.140 (0.134 - 0.144) | 0.000 (-0.057 - 0.059) | 0.000 (-0.059 - 0.058) | 1.003 (0.926 - 1.088) | 1.002 (0.927 - 1.092) |
| 6 | 0.139 (0.133 - 0.144) | 0.139 (0.133 - 0.144) | 0.000 (-0.063 - 0.067) | 0.000 (-0.064 - 0.068) | 1.000 (0.920 - 1.105) | 0.999 (0.914 - 1.099) |
| 7 | 0.138 (0.132 - 0.144) | 0.137 (0.131 - 0.143) | 0.000 (-0.074 - 0.070) | 0.000 (-0.076 - 0.071) | 1.002 (0.908 - 1.115) | 1.003 (0.903 - 1.113) |
| ICU mortality | | | |  |  |  |
| **ICU day** | **SOFA-2**  **Brier (95% CI)** | **SOFA-1**  **Brier (95% CI)** | **SOFA-2**  **intercept (95% CI)** | **SOFA-1**  **intercept (95% CI)** | **SOFA-2**  **slope**  **(95% CI)** | **SOFA-1**  **slope**  **(95% CI)** |
| 1 | 0.070 (0.068 - 0.072) | 0.068 (0.066 - 0.071) | -0.001 (-0.045 - 0.044) | -0.001 (-0.045 - 0.043) | 1.001 (0.969 - 1.036) | 1.001 (0.967 - 1.037) |
| 2 | 0.084 (0.081 - 0.087) | 0.082 (0.079 - 0.085) | -0.001 (-0.052 - 0.051) | -0.001 (-0.051 - 0.052) | 1.001 (0.955 - 1.048) | 1.001 (0.957 - 1.049) |
| 3 | 0.093 (0.090 - 0.097) | 0.092 (0.089 - 0.096) | 0.000 (-0.056 - 0.059) | 0.000 (-0.056 - 0.058) | 1.001 (0.940 - 1.063) | 1.001 (0.943 - 1.057) |
| 4 | 0.095 (0.090 - 0.099) | 0.094 (0.090 - 0.099) | 0.001 (-0.065 - 0.066) | 0.000 (-0.065 - 0.067) | 1.002 (0.936 - 1.080) | 1.002 (0.934 - 1.079) |
| 5 | 0.096 (0.091 - 0.101) | 0.096 (0.091 - 0.101) | -0.001 (-0.074 - 0.072) | -0.001 (-0.074 - 0.074) | 1.000 (0.922 - 1.092) | 1.001 (0.924 - 1.086) |
| 6 | 0.095 (0.089 - 0.101) | 0.095 (0.090 - 0.101) | 0.001 (-0.081 - 0.086) | 0.001 (-0.082 - 0.080) | 1.003 (0.920 - 1.102) | 1.003 (0.917 - 1.099) |
| 7 | 0.097 (0.091 - 0.103) | 0.097 (0.091 - 0.102) | 0.002 (-0.085 - 0.090) | 0.002 (-0.083 - 0.086) | 1.002 (0.901 - 1.110) | 1.001 (0.907 - 1.109) |
| Brier, Intercept and Slope optimism corrected, and 95% CIs computed using bootstrap resampling. | | | | | | |

| ICU day | Number of patients | Dead at 30 days | SOFA-2 AUROC (95% CI) | SOFA-1 AUROC (95% CI) | P value |
| --- | --- | --- | --- | --- | --- |
| 1 | 20221 | 3830 | 0.77 (0.76 - 0.78) | 0.77 (0.76 - 0.78) | p = 1.000 |
| 2 | 14756 | 2956 | 0.75 (0.74 - 0.76) | 0.76 (0.75 - 0.77) | p = 0.004 |
| 3 | 10965 | 2238 | 0.73 (0.72 - 0.74) | 0.73 (0.72 - 0.74) | p = 1.000 |
| 4 | 8316 | 1668 | 0.71 (0.70 - 0.73) | 0.71 (0.70 - 0.73) | p = 1.000 |
| 5 | 6713 | 1321 | 0.70 (0.68 - 0.71) | 0.69 (0.68 - 0.71) | p = 1.000 |
| 6 | 5556 | 1074 | 0.69 (0.67 - 0.71) | 0.69 (0.67 - 0.71) | p = 1.000 |
| 7 | 4735 | 885 | 0.69 (0.68 - 0.71) | 0.69 (0.67 - 0.71) | p = 0.788 |

## **Table S5.** Complete case analysis. Discrimination for SOFA-2 and SOFA-1 at each ICU day.


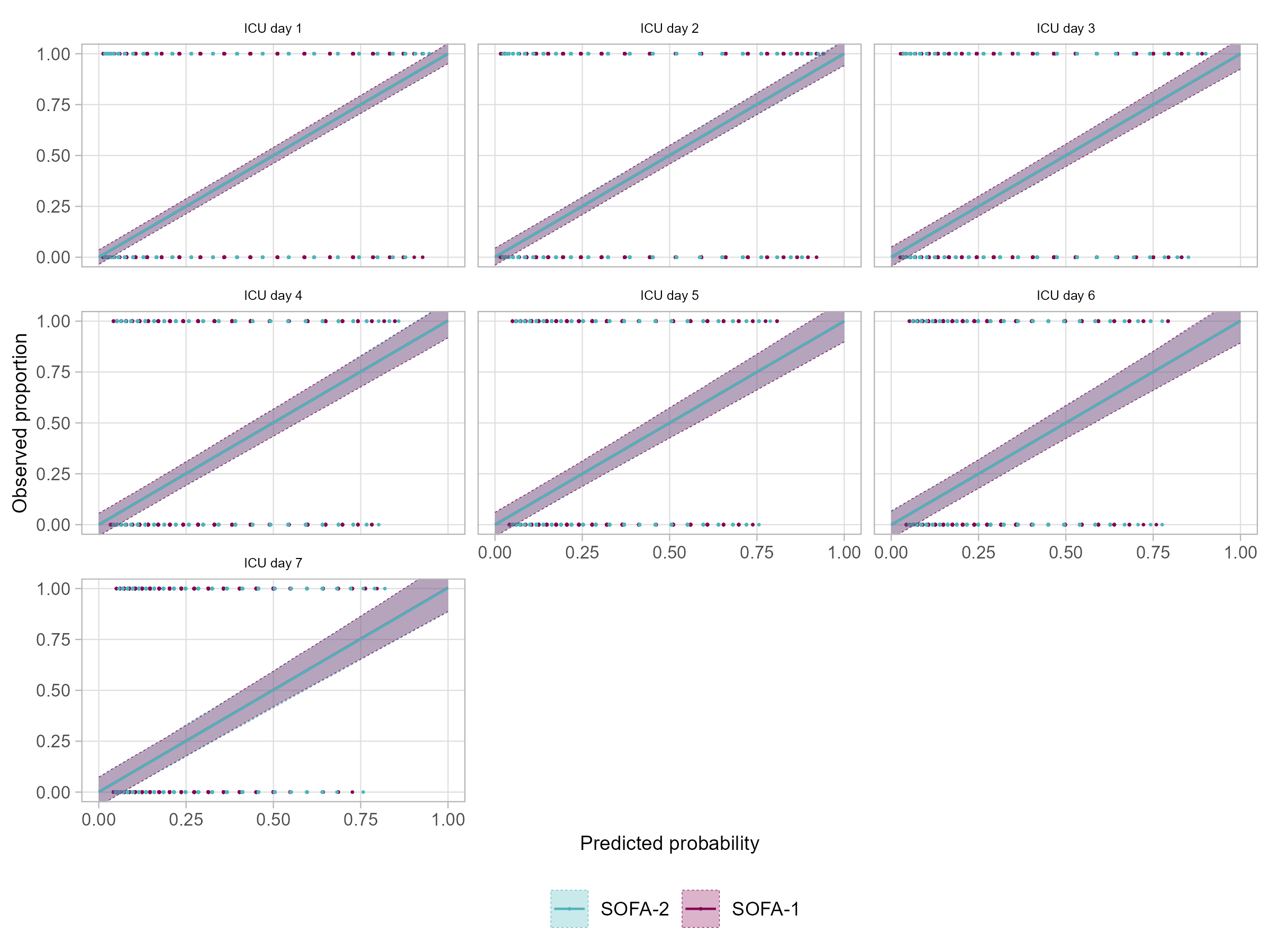


## **Figure S4:** Calibration plot for the univariate logistic regression models with SOFA-2 or SOFA-1 as predictors and 30-day mortality as outcome. Dots: Predicted probability and observed outcome (0 = alive at 30 days, 1 = dead at 30 days) ; Lines: Calibration line with optimism correction; Shaded areas and dotted lines : 95% confidence intervals for calibration line from bootstrap resampling.


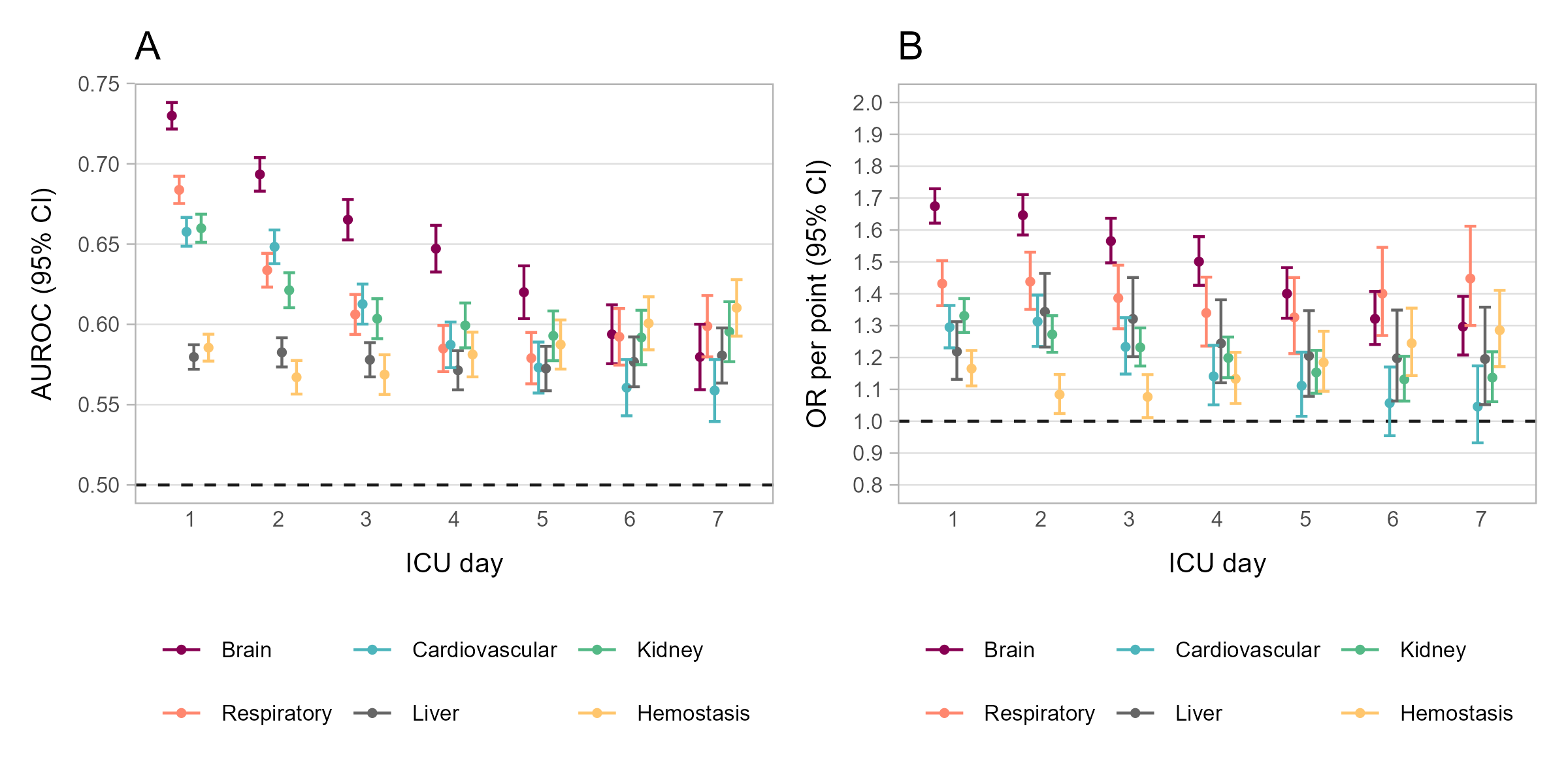


## Figure **S5.** Daily AUROC per SOFA-2 subscore (A) and adjusted OR per 1-unit increase in subscore (B) on 30-day mortality. OR with cluster adjusted confidence intervals and Bonferroni correction. Abbreviations: AUROC area under the receiver operating characteristic curve. OR odds ratio. ICU intensive care unit. CI confidence interval.

| ICU day | SOFA-1 Brier | SOFA-1 Intercept | SOFA-1 Slope | SOFA-2 Brier | SOFA-2 Intercept | SOFA-2 Slope |
| --- | --- | --- | --- | --- | --- | --- |
| 1 | 0.12964 (0.12656 - 0.13237) | -0.000186 (-0.03710 - 0.04100) | 0.9994 (0.9616 - 1.037) | 0.13109 (0.12799 - 0.13385) | -0.000120 (-0.03772 - 0.03976) | 0.9997 (0.9611 - 1.037) |
| 2 | 0.13893 (0.13525 - 0.14222) | 0.001084 (-0.04112 - 0.04604) | 1.0011 (0.9540 - 1.052) | 0.14197 (0.13836 - 0.14533) | 0.000911 (-0.04302 - 0.04455) | 1.0010 (0.9519 - 1.051) |
| 3 | 0.14701 (0.14281 - 0.15123) | -0.000053 (-0.05101 - 0.05114) | 1.0007 (0.9447 - 1.065) | 0.14824 (0.14392 - 0.15249) | -0.000077 (-0.05083 - 0.04965) | 1.0004 (0.9443 - 1.066) |
| 4 | 0.14820 (0.14285 - 0.15285) | 0.000871 (-0.05632 - 0.05750) | 0.9998 (0.9296 - 1.075) | 0.14929 (0.14414 - 0.15397) | 0.000816 (-0.05534 - 0.05928) | 1.0001 (0.9302 - 1.074) |
| 5 | 0.14795 (0.14219 - 0.15323) | -0.000633 (-0.06596 - 0.06491) | 1.0005 (0.9211 - 1.090) | 0.14862 (0.14274 - 0.15390) | -0.000837 (-0.06528 - 0.06297) | 1.0012 (0.9162 - 1.104) |
| 6 | 0.14592 (0.13927 - 0.15166) | 0.000634 (-0.07221 - 0.07707) | 0.9998 (0.9055 - 1.108) | 0.14649 (0.13968 - 0.15246) | 0.000561 (-0.07124 - 0.07405) | 0.9999 (0.9051 - 1.113) |
| 7 | 0.14131 (0.13414 - 0.14803) | -0.000575 (-0.08046 - 0.08491) | 0.9997 (0.9031 - 1.111) | 0.14128 (0.13384 - 0.14776) | -0.000795 (-0.08040 - 0.08791) | 0.9987 (0.9030 - 1.107) |

## **Table S6.** Complete case analysis. Calibration data for SOFA-2 and SOFA-1 at each ICU day.

## **Table S7**. ROC-areas per SOFA-2 component for 30-day mortality

| **ICU day** | **Brain** | **Respiratory** | **Cardiovascular** | **Liver** | **Kidney** | **Hemostasis** |
| --- | --- | --- | --- | --- | --- | --- |
| 1 | 0.73 (0.72 - 0.74) | 0.66 (0.65 - 0.67) | 0.59 (0.58 - 0.59) | 0.66 (0.65 - 0.67) | 0.58 (0.57 - 0.59) | 0.68 (0.68 - 0.69) |
| 2 | 0.69 (0.68 - 0.70) | 0.65 (0.64 - 0.66) | 0.57 (0.56 - 0.58) | 0.62 (0.61 - 0.63) | 0.58 (0.57 - 0.59) | 0.63 (0.62 - 0.64) |
| 3 | 0.67 (0.65 - 0.68) | 0.61 (0.60 - 0.63) | 0.57 (0.56 - 0.58) | 0.60 (0.59 - 0.62) | 0.58 (0.57 - 0.59) | 0.61 (0.59 - 0.62) |
| 4 | 0.65 (0.63 - 0.66) | 0.59 (0.57 - 0.60) | 0.58 (0.57 - 0.60) | 0.60 (0.59 - 0.61) | 0.57 (0.56 - 0.58) | 0.58 (0.57 - 0.60) |
| 5 | 0.62 (0.60 - 0.64) | 0.57 (0.56 - 0.59) | 0.59 (0.57 - 0.60) | 0.59 (0.58 - 0.61) | 0.57 (0.56 - 0.59) | 0.58 (0.56 - 0.59) |
| 6 | 0.59 (0.58 - 0.61) | 0.56 (0.54 - 0.58) | 0.60 (0.58 - 0.62) | 0.59 (0.57 - 0.61) | 0.58 (0.56 - 0.59) | 0.59 (0.57 - 0.61) |
| 7 | 0.58 (0.56 - 0.60) | 0.56 (0.54 - 0.58) | 0.61 (0.59 - 0.63) | 0.60 (0.58 - 0.61) | 0.58 (0.56 - 0.60) | 0.60 (0.58 - 0.62) |

## **Table S8**. Conditional OR per point per domain from generalized mixed-effect model with binomial link and patient-level random intercept.

| **Subscore** | **OR (95% CI)** | **p-value** |
| --- | --- | --- |
| Brain | 1.32 (1.18 - 1.48) | p < 0.001 |
| Respiratory | 1.07 (0.91 - 1.26) | p = 0.405 |
| Cardiovascular | 0.93 (0.79 - 1.09) | p = 0.370 |
| Liver | 1.23 (0.97 - 1.57) | p = 0.088 |
| Kidney | 1.22 (1.07 - 1.39) | p = 0.003 |
| Hemostasis | 1.13 (0.96 - 1.33) | p = 0.128 |

## **Table S9a**. AUROC of delta SOFA-2 and delta SOFA-1 for 30-day mortality

| **∆SOFA** | **No. of admissions** | **∆SOFA-2**  **AUROC (95% CI)** | **∆SOFA-1**  **AUROC (95% CI)** | **p-value** |
| --- | --- | --- | --- | --- |
| D2-D1 | 17803 | 0.52 (0.51 - 0.54) | 0.53 (0.52 - 0.54) | 1 |
| D3-D1 | 12389 | 0.54 (0.53 - 0.56) | 0.54 (0.53 - 0.55) | 1 |
| D4-D1 | 9611 | 0.55 (0.53 - 0.56) | 0.55 (0.53 - 0.56) | 1 |
| D5-D1 | 7706 | 0.55 (0.53 - 0.56) | 0.55 (0.53 - 0.56) | 1 |
| D6-D1 | 6344 | 0.56 (0.54 - 0.58) | 0.55 (0.53 - 0.57) | 1 |
| D7-D1 | 5347 | 0.57 (0.55 - 0.59) | 0.56 (0.54 - 0.58) | 0.585 |
| D3-D2 | 12389 | 0.54 (0.53 - 0.56) | 0.54 (0.53 - 0.55) | 1 |
| D4-D3 | 9611 | 0.55 (0.53 - 0.56) | 0.55 (0.54 - 0.57) | 1 |
| D5-D4 | 7706 | 0.54 (0.53 - 0.56) | 0.54 (0.52 - 0.55) | 1 |
| D6-D5 | 6344 | 0.54 (0.53 - 0.56) | 0.55 (0.53 - 0.57) | 1 |
| D7-D6 | 5347 | 0.55 (0.53 - 0.57) | 0.55 (0.53 - 0.57) | 1 |
| AUROC, area under the receiver operating characteristic curve  P-values represents the comparison between **∆**SOFA-2 and **∆**SOFA-1 on each day and were Bonferroni-corrected for multiple comparisons. | | | | |

## **Table S9b**. AUROC of delta SOFA-2 and delta SOFA-1 for ICU mortality

| **∆SOFA** | **No. of admissions** | **∆SOFA-2**  **AUROC (95% CI)** | **∆SOFA-1**  **AUROC (95% CI)** | **p-value** |
| --- | --- | --- | --- | --- |
| D2-D1 | 17803 | 0.56 (0.55 - 0.58) | 0.57 (0.56 - 0.59) | 0.263 |
| D3-D1 | 12389 | 0.59 (0.58 - 0.61) | 0.60 (0.58 - 0.61) | 1 |
| D4-D1 | 9611 | 0.59 (0.57 - 0.61) | 0.60 (0.58 - 0.62) | 1 |
| D5-D1 | 7706 | 0.60 (0.58 - 0.62) | 0.61 (0.59 - 0.63) | 1 |
| D6-D1 | 6344 | 0.62 (0.60 - 0.64) | 0.62 (0.60 - 0.64) | 1 |
| D7-D1 | 5347 | 0.63 (0.61 - 0.66) | 0.62 (0.60 - 0.65) | 1 |
| D3-D2 | 12389 | 0.57 (0.56 - 0.59) | 0.57 (0.55 - 0.58) | 1 |
| D4-D3 | 9611 | 0.56 (0.55 - 0.58) | 0.57 (0.55 - 0.59) | 1 |
| D5-D4 | 7706 | 0.58 (0.56 - 0.60) | 0.57 (0.56 - 0.59) | 1 |
| D6-D5 | 6344 | 0.57 (0.55 - 0.59) | 0.58 (0.55 - 0.60) | 1 |
| D7-D6 | 5347 | 0.56 (0.54 - 0.59) | 0.55 (0.53 - 0.58) | 1 |
| AUROC, area under the receiver operating characteristic curve  P-values represents the comparison between **∆**SOFA-2 and **∆**SOFA-1 on each day and were Bonferroni-corrected for multiple comparisons. | | | | |

## **Table S10**. Characteristics of ICU patients admitted due to trauma or sepsis

|  | **Trauma** | **Sepsis** | **p-value** |
| --- | --- | --- | --- |
|  | **N=2,982** | **N=5,206** |  |
| Age, mean (SD), year | 50 (20) | 61 (15) | <0.001 |
| Male sex, n (%) | 2,214 (74.2%) | 3,262 (62.7%) | <0.001 |
| Charlson comorbidity index, median (IQR) | 0 (0-1) | 3 (1-5) | <0.001 |
| Selected comorbidities, n (%) |  |  |  |
| Renal disease | 105 ( 3.5%) | 899 (17.3%) | <0.001 |
| Liver disease | 167 ( 5.6%) | 640 (12.3%) | <0.001 |
| Cardiovascular disease | 487 (16.3%) | 2,105 (40.4%) | <0.001 |
| Pulmonary disease | 291 ( 9.8%) | 1,186 (22.8%) | <0.001 |
| Malignancy | 177 ( 5.9%) | 1,739 (33.4%) | <0.001 |
| Invasive mechanical ventilation at ICU admission, n (%) | 1,850 (62.0%) | 1,996 (38.3%) | <0.001 |
| ICU length of stay, median (IQR), days | 2 (1-6) | 3 (1-7) | <0.001 |
| ICU mortality, n (%) | 307 (10.3%) | 657 (12.6%) | 0.002 |
| 30-day mortality, n (%) | 449 (15.1%) | 1,131 (21.7%) | <0.001 |

## **Table** S11a. AUROC for SOFA-2 and SOFA-1 for 30-day mortality in patients with sepsis.

| **ICU day** | **No. of deaths/**  **No. of admissions** | **SOFA-2 AUROC**  **(95% CI)** | **SOFA-1 AUROC**  **(95% CI)** | **p-value** |
| --- | --- | --- | --- | --- |
| 1 | 1131/5206 | 0.72 (0.70 - 0.74) | 0.72 (0.70 - 0.74) | p = 1.000 |
| 2 | 968/4187 | 0.73 (0.71 - 0.75) | 0.73 (0.71 - 0.75) | p = 1.000 |
| 3 | 760/3245 | 0.71 (0.69 - 0.73) | 0.72 (0.70 - 0.74) | p = 1.000 |
| 4 | 579/2553 | 0.69 (0.67 - 0.72) | 0.71 (0.69 - 0.73) | p = 0.021 |
| 5 | 467/2068 | 0.69 (0.66 - 0.71) | 0.69 (0.66 - 0.72) | p = 1.000 |
| 6 | 381/1709 | 0.68 (0.65 - 0.71) | 0.69 (0.66 - 0.72) | p = 1.000 |
| 7 | 327/1458 | 0.68 (0.65 - 0.72) | 0.70 (0.67 - 0.73) | p = 0.562 |

## **Table S11b**. Calibration metrics for SOFA-2 and SOFA-1 for 30-day mortality in patients with sepsis.

| **ICU day** | **SOFA-2**  **Brier (95% CI)** | **SOFA-1**  **Brier**  **(95% CI)** | **SOFA-2**  **intercept**  **(95% CI)** | **SOFA-1**  **intercept**  **(95% CI)** | **SOFA-2**  **slope**  **(95% CI)** | **SOFA-1**  **slope**  **(95% CI)** |
| --- | --- | --- | --- | --- | --- | --- |
| 1 | 0.151 (0.145 - 0.157) | 0.150 (0.144 - 0.156) | 0.000 (-0.073 - 0.068) | 0.000 (-0.070 - 0.067) | 0.999 (0.918 - 1.093) | 1.000 (0.918 - 1.093) |
| 2 | 0.157 (0.150 - 0.163) | 0.154 (0.147 - 0.160) | -0.001 (-0.079 - 0.077) | -0.001 (-0.077 - 0.076) | 0.999 (0.914 - 1.090) | 0.999 (0.909 - 1.093) |
| 3 | 0.162 (0.155 - 0.170) | 0.160 (0.153 - 0.168) | 0.000 (-0.085 - 0.085) | 0.000 (-0.085 - 0.083) | 1.005 (0.894 - 1.134) | 1.005 (0.904 - 1.136) |
| 4 | 0.162 (0.152 - 0.169) | 0.160 (0.150 - 0.167) | 0.003 (-0.095 - 0.103) | 0.003 (-0.097 - 0.106) | 1.001 (0.869 - 1.152) | 1.002 (0.874 - 1.144) |
| 5 | 0.161 (0.151 - 0.170) | 0.160 (0.150 - 0.168) | 0.004 (-0.099 - 0.107) | 0.004 (-0.103 - 0.110) | 1.009 (0.868 - 1.198) | 1.008 (0.866 - 1.170) |
| 6 | 0.161 (0.148 - 0.170) | 0.159 (0.146 - 0.168) | 0.004 (-0.122 - 0.128) | 0.003 (-0.119 - 0.124) | 1.007 (0.848 - 1.200) | 1.007 (0.855 - 1.190) |
| 7 | 0.160 (0.148 - 0.171) | 0.157 (0.144 - 0.167) | 0.003 (-0.130 - 0.130) | 0.003 (-0.130 - 0.133) | 1.006 (0.835 - 1.218) | 1.004 (0.845 - 1.183) |

## **Table S12a**. AUROC for SOFA-2 and SOFA-1 for 30-day mortality in trauma patients.

| **ICU day** | **No. of deaths/**  **No. of admissions** | **SOFA-2 AUROC**  **(95% CI)** | **SOFA-1 AUROC**  **(95% CI)** | **p-value** |
| --- | --- | --- | --- | --- |
| 1 | 449/2982 | 0.80 (0.78 - 0.83) | 0.81 (0.79 - 0.83) | p = 1.000 |
| 2 | 328/1922 | 0.78 (0.75 - 0.81) | 0.79 (0.77 - 0.82) | p = 0.341 |
| 3 | 224/1431 | 0.77 (0.73 - 0.80) | 0.75 (0.72 - 0.78) | p = 0.372 |
| 4 | 157/1124 | 0.73 (0.69 - 0.77) | 0.71 (0.67 - 0.75) | p = 0.627 |
| 5 | 121/917 | 0.70 (0.66 - 0.75) | 0.67 (0.63 - 0.72) | p = 0.305 |
| 6 | 90/793 | 0.71 (0.66 - 0.76) | 0.68 (0.63 - 0.74) | p = 0.516 |
| 7 | 72/699 | 0.73 (0.67 - 0.79) | 0.69 (0.63 - 0.76) | p = 0.288 |

## **Table S12b**. Calibration metrics for SOFA-2 and SOFA-1 for 30-day mortality in patients with trauma.

| **ICU day** | **SOFA-2**  **Brier (95% CI)** | **SOFA-1**  **Brier**  **(95% CI)** | **SOFA-2**  **intercept**  **(95% CI)** | **SOFA-1**  **intercept**  **(95% CI)** | **SOFA-2**  **slope**  **(95% CI)** | **SOFA-1**  **slope**  **(95% CI)** |
| --- | --- | --- | --- | --- | --- | --- |
| 1 | 0.106 (0.097 - 0.114) | 0.105 (0.097 - 0.112) | 0.000 (-0.123 - 0.117) | 0.000 (-0.124 - 0.113) | 0.999 (0.897 - 1.103) | 0.999 (0.902 - 1.103) |
| 2 | 0.120 (0.110 - 0.129) | 0.117 (0.108 - 0.126) | 0.003 (-0.123 - 0.139) | 0.003 (-0.125 - 0.132) | 1.001 (0.889 - 1.132) | 0.999 (0.882 - 1.127) |
| 3 | 0.116 (0.104 - 0.126) | 0.118 (0.106 - 0.128) | 0.005 (-0.148 - 0.158) | 0.004 (-0.149 - 0.159) | 1.006 (0.853 - 1.156) | 1.001 (0.854 - 1.162) |
| 4 | 0.113 (0.097 - 0.125) | 0.112 (0.096 - 0.124) | 0.003 (-0.172 - 0.203) | 0.004 (-0.175 - 0.204) | 0.999 (0.809 - 1.251) | 0.997 (0.816 - 1.250) |
| 5 | 0.110 (0.093 - 0.124) | 0.109 (0.092 - 0.123) | 0.005 (-0.188 - 0.223) | 0.006 (-0.187 - 0.218) | 1.017 (0.785 - 1.342) | 1.021 (0.775 - 1.392) |
| 6 | 0.098 (0.081 - 0.113) | 0.097 (0.080 - 0.112) | 0.001 (-0.220 - 0.244) | 0.002 (-0.218 - 0.247) | 1.015 (0.764 - 1.415) | 1.023 (0.746 - 1.474) |
| 7 | 0.089 (0.070 - 0.104) | 0.089 (0.069 - 0.104) | 0.007 (-0.237 - 0.283) | 0.009 (-0.229 - 0.294) | 1.013 (0.752 - 1.379) | 1.023 (0.723 - 1.416) |

## **Table S13.** Baseline characteristics of ICU admissions with SOFA-1 score ≥10 on ICU arrival.

|  | **SOFA-1 ≥ 10** |
| --- | --- |
| **Characteristic** | **N=3,635** |
| Age, median (IQR), year | 66 (55-73) |
| Male sex, n (%) | 2,428 (66.8%) |
| Charlson comorbidity index, median (IQR) | 3 (1-5) |
| Selected comorbidities, n (%) |  |
| Renal disease | 864 (23.8%) |
| Liver disease | 660 (18.2%) |
| Cardiovascular disease | 1,556 (42.8%) |
| Pulmonary disease | 748 (20.6%) |
| Malignancy | 1,166 (32.1%) |
| Invasive mechanical ventilation at ICU admission, n (%) | 2,279 (62.7%) |
| SOFA-1 score ICU-day 1, median (IQR) | 13 (11-14) |
| ICU length of stay, median (IQR), days | 3 (1-8) |
| ICU mortality, n (%) | 1,148 (31.6%) |
| 30-day mortality, n (%) | 1,626 (44.7%) |

## **Table** S14a. AUROC for SOFA-2 and SOFA-1 for 30-day mortality in patients with SOFA-1 ≥10 at admission

| **ICU day** | **No. of deaths/**  **No. of admissions** | **SOFA-2 AUROC**  **(95% CI)** | **SOFA-1 AUROC**  **(95% CI)** | **p-value** |
| --- | --- | --- | --- | --- |
| 1 | 1626/3635 | 0.62 (0.60 - 0.64) | 0.64 (0.62 - 0.66) | p = 0.023 |
| 2 | 1226/3010 | 0.65 (0.63 - 0.67) | 0.67 (0.65 - 0.69) | p = 0.001 |
| 3 | 906/2367 | 0.64 (0.61 - 0.66) | 0.64 (0.62 - 0.67) | p = 0.583 |
| 4 | 689/1919 | 0.62 (0.59 - 0.65) | 0.63 (0.61 - 0.66) | p = 0.320 |
| 5 | 529/1583 | 0.62 (0.59 - 0.65) | 0.64 (0.61 - 0.67) | p = 0.061 |
| 6 | 431/1357 | 0.62 (0.59 - 0.66) | 0.64 (0.61 - 0.67) | p = 0.578 |
| 7 | 346/1161 | 0.64 (0.61 - 0.68) | 0.66 (0.63 - 0.70) | p = 0.293 |

## **Table S14b**. Calibration metrics for SOFA-2 and SOFA-1 for 30-day mortality in patients with SOFA-1 ≥10 at admission

| **ICU day** | **SOFA-2**  **Brier (95% CI)** | **SOFA-1**  **Brier**  **(95% CI)** | **SOFA-2**  **intercept**  **(95% CI)** | **SOFA-1**  **intercept**  **(95% CI)** | **SOFA-2**  **slope**  **(95% CI)** | **SOFA-1**  **slope**  **(95% CI)** |
| --- | --- | --- | --- | --- | --- | --- |
| 1 | 0.235 (0.231 - 0.238) | 0.232 (0.228 - 0.235) | 0.000 (-0.070 - 0.067) | 0.000 (-0.072 - 0.068) | 1.003 (0.866 - 1.155) | 1.002 (0.887 - 1.140) |
| 2 | 0.225 (0.219 - 0.229) | 0.221 (0.215 - 0.225) | 0.001 (-0.074 - 0.076) | 0.001 (-0.076 - 0.074) | 1.005 (0.883 - 1.167) | 1.004 (0.892 - 1.144) |
| 3 | 0.223 (0.216 - 0.228) | 0.222 (0.215 - 0.227) | -0.001 (-0.091 - 0.088) | -0.001 (-0.089 - 0.085) | 1.009 (0.858 - 1.184) | 1.008 (0.864 - 1.182) |
| 4 | 0.220 (0.213 - 0.226) | 0.218 (0.211 - 0.224) | -0.001 (-0.095 - 0.089) | -0.001 (-0.094 - 0.088) | 1.016 (0.833 - 1.283) | 1.015 (0.841 - 1.243) |
| 5 | 0.213 (0.203 - 0.220) | 0.210 (0.200 - 0.217) | 0.001 (-0.104 - 0.107) | 0.002 (-0.105 - 0.109) | 1.020 (0.803 - 1.314) | 1.017 (0.831 - 1.261) |
| 6 | 0.207 (0.196 - 0.215) | 0.204 (0.193 - 0.212) | 0.000 (-0.117 - 0.107) | -0.001 (-0.118 - 0.111) | 1.012 (0.796 - 1.322) | 1.011 (0.819 - 1.280) |
| 7 | 0.196 (0.182 - 0.205) | 0.192 (0.178 - 0.201) | 0.002 (-0.126 - 0.136) | 0.002 (-0.126 - 0.134) | 1.010 (0.815 - 1.271) | 1.009 (0.827 - 1.255) |


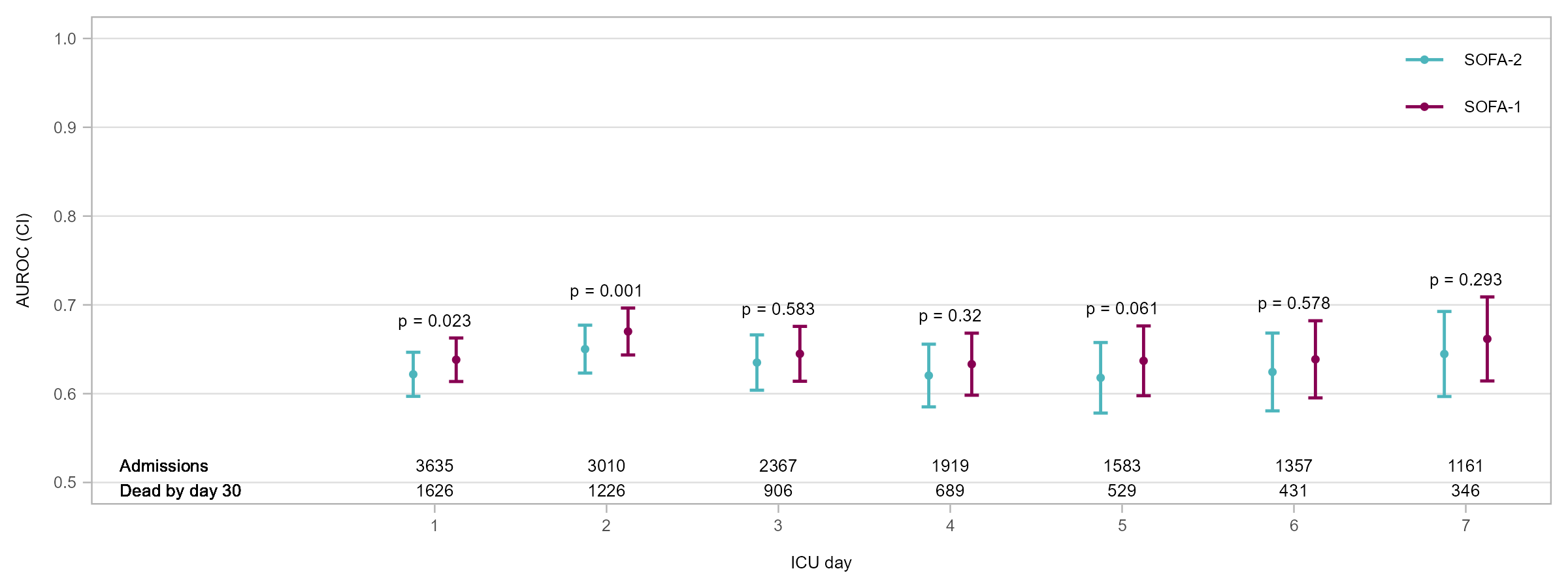


## **Figure S6.** AUROCs with 95% CI for patients admitted with SOFA-1 score ≥10 (A)

| SOFA 1 | | | | | | |
| --- | --- | --- | --- | --- | --- | --- |
| ICU day | Central nervous system | Respiratory | Circulatory | Liver | Renal | Coagulation |
| 1 | 3060 (10.26%) | 95 (0.32%) | 2993 (10.04%) | 8266 (27.72%) | 147 (0.49%) | 4240 (14.22%) |
| 2 | 924 (5.19%) | 18 (0.1%) | 1314 (7.38%) | 2168 (12.18%) | 29 (0.16%) | 1130 (6.35%) |
| 3 | 1397 (11.28%) | 110 (0.89%) | 863 (6.97%) | 863 (6.97%) | 70 (0.57%) | 536 (4.33%) |
| 4 | 1073 (11.16%) | 114 (1.19%) | 767 (7.98%) | 568 (5.91%) | 73 (0.76%) | 400 (4.16%) |
| 5 | 787 (10.21%) | 114 (1.48%) | 699 (9.07%) | 397 (5.15%) | 67 (0.87%) | 290 (3.76%) |
| 6 | 595 (9.38%) | 108 (1.7%) | 561 (8.84%) | 280 (4.41%) | 66 (1.04%) | 218 (3.44%) |
| 7 | 465 (8.7%) | 93 (1.74%) | 436 (8.15%) | 249 (4.66%) | 60 (1.12%) | 190 (3.55%) |

## Table S15a. Missing per domain SOFA-1.

| SOFA 2 | | | | | | |
| --- | --- | --- | --- | --- | --- | --- |
| ICU day | Brain | Respiration | Cardiovascular | Liver | Kidney | Hemostasis |
| 1 | 2861 (9.59%) | 95 (0.32%) | 2912 (9.77%) | 8266 (27.72%) | 121 (0.41%) | 4240 (14.22%) |
| 2 | 817 (4.59%) | 18 (0.1%) | 1222 (6.86%) | 2168 (12.18%) | 17 (0.1%) | 1130 (6.35%) |
| 3 | 1250 (10.09%) | 110 (0.89%) | 796 (6.43%) | 863 (6.97%) | 26 (0.21%) | 536 (4.33%) |
| 4 | 943 (9.81%) | 114 (1.19%) | 704 (7.32%) | 568 (5.91%) | 28 (0.29%) | 400 (4.16%) |
| 5 | 685 (8.89%) | 114 (1.48%) | 640 (8.31%) | 397 (5.15%) | 28 (0.36%) | 290 (3.76%) |
| 6 | 513 (8.09%) | 108 (1.7%) | 513 (8.09%) | 280 (4.41%) | 27 (0.43%) | 218 (3.44%) |
| 7 | 391 (7.31%) | 93 (1.74%) | 398 (7.44%) | 249 (4.66%) | 22 (0.41%) | 190 (3.55%) |

## Table S15b. Missing per domain SOFA-2.

| **Subscore** | **Agreement of organ failure (Score ≥2)** |
| --- | --- |
| Brain | 98.78% |
| Cardiovascular | 99.39% |
| Hemostasis | 99.97% |
| Kidney | 88.05% |
| Liver | 94.93% |
| Respiratory | 78.36% |

## Table S16. Agreement of failure (score ≥2) per domain between SOFA-2 and SOFA-1 on ICU day 1.
